# Monitoring TRPC7 Conformational Changes by BRET Following GPCR Activation

**DOI:** 10.3390/ijms23052502

**Published:** 2022-02-24

**Authors:** Cécile Pétigny, Audrey-Ann Dumont, Hugo Giguère, Audrey Collette, Brian J. Holleran, Mircea Iftinca, Christophe Altier, Élie Besserer-Offroy, Mannix Auger-Messier, Richard Leduc

**Affiliations:** 1 Department of Pharmacology-Physiology, Faculty of Medicine and Health Sciences, Université de Sherbrooke, Sherbrooke, QC J1H 5N4, Canada; cecile.petigny@usherbrooke.ca (C.P.); audrey.collette@usherbrooke.ca (A.C.); brian.holleran@usherbrooke.ca (B.J.H.); 2 Institut de Pharmacologie de Sherbrooke, Université de Sherbrooke, Sherbrooke, QC J1H 5N4, Canada; audrey-ann.dumont@usherbrooke.ca (A.-A.D.); hugo.giguere@usherbrooke.ca (H.G.); mannix.auger-messier@usherbrooke.ca (M.A.-M.); 3 Centre de Recherche du Centre Hospitalier Universitaire de Sherbrooke, Sherbrooke, QC J1H 5N4, Canada; 4 Department of Medicine, Division of Cardiology, Faculty of Medicine and Health Sciences, Université de Sherbrooke, Sherbrooke, QC J1H 5N4, Canada; 5 Department of Physiology and Pharmacology, Inflammation Research Network-Snyder Institute for Chronic Diseases and Alberta Children’s Hospital Research Institute, University of Calgary, Calgary, AB T2N 1N4, Canada; miftinca@ucalgary.ca (M.I.); altier@ucalgary.ca (C.A.); 6 Department of Molecular and Medical Pharmacology, Ahmanson Translational Theranostics Division, David Geffen School of Medicine, University of California-Los Angeles, Los Angeles, CA 90095, USA; ebesserer@ucla.edu; 7 Jonsson Comprehensive Cancer Center, University of California, Los Angeles, CA 90095, USA; 8 California NanoSystems Institute, Los Angeles, CA 90095, USA

**Keywords:** transient receptor potential canonical (TRPC), G protein-coupled receptor (GPCR), bioluminescence resonance energy transfer (BRET), Angiotensin II (AngII), Angiotensin II type-1 receptor (AT_1_R), G protein alpha q subunit, cellular signaling

## Abstract

Transient receptor potential canonical (TRPC) channels are membrane proteins involved in regulating Ca^2+^ homeostasis, and whose functions are modulated by G protein-coupled receptors (GPCR). In this study, we developed bioluminescent resonance energy transfer (BRET) biosensors to better study channel conformational changes following receptor activation. For this study, two intramolecular biosensors, GFP10-TRPC7-RLucII and RLucII-TRPC7-GFP10, were constructed and were assessed following the activation of various GPCRs. We first transiently expressed receptors and the biosensors in HEK293 cells, and BRET levels were measured following agonist stimulation of GPCRs. The activation of GPCRs that engage Gα_q_ led to a Gα_q_-dependent BRET response of the functional TRPC7 biosensor. Focusing on the Angiotensin II type-1 receptor (AT_1_R), GFP10-TRPC7-RLucII was tested in rat neonatal cardiac fibroblasts, expressing endogenous AT_1_R and TRPC7. We detected similar BRET responses in these cells, thus validating the use of the biosensor in physiological conditions. Taken together, our results suggest that activation of Gα_q_-coupled receptors induce conformational changes in a novel and functional TRPC7 BRET biosensor.

## 1. Introduction

Transient Receptor Potential (TRP) is a superfamily of transmembrane proteins that function as ion channels. TRPC (“C” for canonical) was the first subfamily identified in the six existing subfamilies, which includes six distinct members: TRPC1,3-7 (*TRPC2* is a pseudogene) [1]. Similar to other TRPs, they are constituted of six transmembrane domains (TM1–TM6), with a pore region between TM5 and TM6 and cytoplasmic N- and C-termini. They also present several motifs, reported to be important for their trafficking and interactions with other proteins or TRPs, such as ankyrin repeat, coil-coiled domains, PSD-95, Discs-large, ZO-1 (PDZ), and calmodulin/IP3 receptor binding (CIRB) domains [2]. TRPC subunits assemble in an oligomeric structure thereby forming homo-tetrameric or hetero-tetrameric non-selective cation channels that play an important role in ion homeostasis. While TRPC channels are reported to be non-selective, they enable Ca^2+^ entry [3]. TRPC proteins can be divided in two groups, depending on their sequence and functional homologies: (i) TRPC1/4/5 are diacylglycerol (DAG)-insensitive and activated by complex pathways [4], and (ii) TRPC3/6/7 are activated by DAG and its analogs [4,5].

TRPC7 is the most recently identified member of the TRPC subfamily. It is abundantly expressed in the brain, lung, and heart [5], and is highly homologous to TRPC3 and TRPC6 (81% and 75% sequence homology, respectively) [6]. It has been described as a nociceptive mechanoreceptor [7], especially activated downstream of Gα_q_-coupled G protein-coupled receptors (GPCR), via the hydrolysis of phosphatidylinositol-4,5-biphosphate (PIP_2_) by phospholipase C (PLC), producing DAG as a second messenger [5]. Although the intricacies of TRPC7 channel activation remain unclear, it is well established that it is directly activated by DAG [4,8,9]. Other studies have also demonstrated that TRPC7 is activated indirectly by DAG through other interacting proteins [10], or independently by PIP_2_ or IP_3_ [11,12].

TRPC7 is implicated in physiological as well as pathological conditions, such as in age-related diseases like UVB-induced skin aging [7], initiation of acute seizure [13], and myocardial apoptosis [14]. Conversely, TRPC7 possesses a potential protective effect in breast cancer [15]. Altered TRPC expression is often associated with the development and progression of cardiac pathologies. TRPC7, along with other members of TRPC family (i.e., TRPC3 and TRPC6) are involved in these pathologies in response to the activation of Angiotensin II type-1 receptor (AT_1_R) [14,16,17]. Thus, several BRET-based biosensors of TRP channels have been successfully developed in the past in order to characterize biological processes associated with these ion channels [18,19]. However, a TRPC7 BRET-based biosensor has never yet been described. In this study, we designed novel intramolecular TRPC7 BRET-based biosensors to characterize TRPC7 conformational changes that occur following stimulation of various GPCRs. These biosensors allowed us to study TRPC7 activation, especially downstream of AT_1_R transiently expressed in HEK293 cells, and endogenously expressed in neonatal rat cardiac fibroblasts.

## 2. Results and Discussion

### 2.1. Design of TRPC7 BRET-Based Biosensors

**Figure 1:**
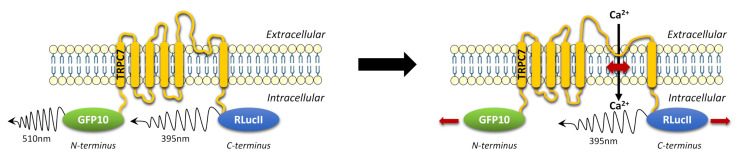
TRPC7 biosensors were designed to detect their conformational changes in a BRET^2^ assay. The double brilliance intramolecular BRET TRPC7 biosensor was double-tagged N-terminally with GFP10 and C-terminally with RLucII (or N-term RLucII and C-term GFP10). TRPC7, Transient receptor potential canonical channel 7; RlucII, *Renilla* luciferase; GFP10, green fluorescent protein.

We developed intramolecular TRPC7 BRET-based biosensors to detect TRPC7 conformational changes with *Renilla* luciferase (RLucII), and a modified version of the green fluorescent protein (GFP10) fused to the N-terminus and the C-terminus, respectively (RLucII-TRPC7-GFP10), or vice versa (GFP10-TRPC7-RlucII) (Figure 1). These constructs are reminiscent of similar intramolecular BRET constructs previously used to examine elements of signaling pathways such as β-arrestin [20].

As Liu et al. developed an intramolecular BRET to monitor TRPC3 activity, we used the same design for TRPC7 [18]. However, while their method of detection was BRET^1^, we used BRET^2^, changing the YFP for a GFP10, thus increasing the wavelength separation between RLucII and fluorescent protein emission, resulting in an improved detection and discrimination of signals [21].

### 2.2. Activation of the Gα_q_ Pathway Results in Conformational Changes of Functional Intramolecular TRPC7 Biosensors

**Figure 2:**
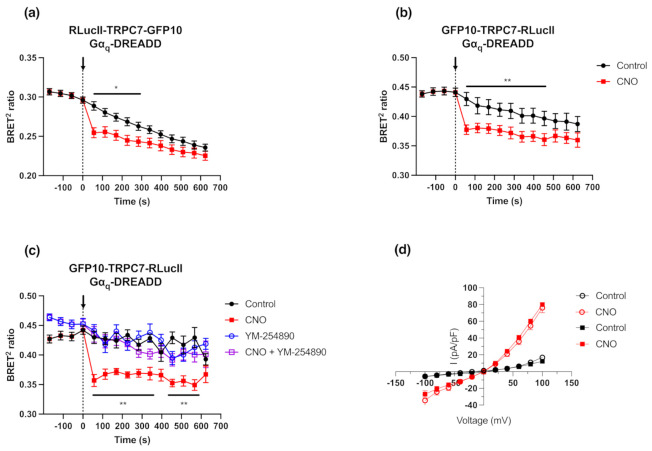
Double brilliance GFP10-TRPC7-RLucII biosensors allow the detection by BRET of TRPC7 conformational changes in response to Gα_q_-DREADD activation while being fully functional. (**a**,**b**) HEK293 cells were co-transfected with plasmids encoding RLucII-TRPC7-GFP10 or GFP10-TRPC7-RLucII biosensors (75 ng) and Gα_q_-DREADD (500 ng), and were stimulated with Clozapine-N-Oxide (CNO; 1 µM) or vehicle as a control. The BRET signal was measured for 10 min. (**c**) HEK293 cells were transfected in the same conditions as in (**a**) and were pre-incubated with YM-254890 (Gα_q_ inhibitor; 1 µM) or vehicle for 10 min before basal BRET signal was read. The BRET signal was measured for 10 min following stimulation with CNO (1 µM). Each data set represents the mean of three independent experiments, which were each performed in triplicate, and expressed as the mean ± S.E.M. (**d**) Whole-cell patch-clamp experiments on HEK293 cells were performed 48 h after co-transfection with plasmids encoding Gα_q_-coupled DREADD and TRPC7 WT (open circles) or the GFP10-TRPC7-RLucII biosensor (closed squares). Current–voltage relationships were obtained from the TRPC7 WT and GFP10-TRPC7-RLucII in control conditions (black symbols) and following Gα_q_-coupled DREADD stimulation with CNO (red symbols; 1 µM). Each data set represents the mean of seven (7) recordings ± S.E.M. Statistical analyses were performed using a two-way ANOVA with multiple comparisons followed by a Sidak’s post-hoc test. * *p* < 0.05, ** *p* < 0.01 for control vs. CNO.

As TRPC7 is activated by downstream Gα_q_ signaling effectors [5], we first examined and compared the BRET signals of RLucII-TRPC7-GFP10 and GFP10-TRPC7-RLucII biosensors in conjunction with the use of Gα_q_ Designer Receptors Exclusively Activated by Designer Drugs (Gα_q_-DREADD), a Gα_q_-biased seven transmembrane synthetic receptor [22]. Co-expression of TRPC7 biosensors with Gα_q_-DREADD and stimulation with 1 µM of Clozapine-N-Oxide (CNO; DREADD’s synthetic and selective agonist) led to a decrease in BRET ratio with both intramolecular biosensor configurations (Figure 2a,b). This observation suggested that a conformational change of the TRPC7 channel, such as distancing or orientation change of the tagged N-terminus and C-terminus, occurs following Gα_q_-DREADD stimulation. An optimal dynamic range was observed with transfection of 25 ng for RLucII-TRPC7-GFP10 (0.022 ± 0.004) and 75 ng for GFP10-TRPC7-RLucII (0.043 ± 0.004), respectively (Figure S1), and was sustained over time (Figure 2a,b). Since the GFP10-TRPC7-RLucII construct possessed the ideal combination of RLucII signal intensity and dynamic range, as well as a stable signal over time, we used this construct in subsequent experiments.

We then verified whether the conformational changes of GFP10-TRPC7-RLucII biosensor could be specifically due to Gα_q_ signaling downstream of Gα_q_-DREADD activation. Pre-treatment with 1 µM of the Gα_q_ inhibitor YM-254890 [23] completely abolished Gα_q_-DREADD-induced TRPC7 conformational changes (Figure 2c). Importantly, we also verified whether GFP10-TRPC7-RLucII maintained the same electrophysiological profile as its wild-type (WT) counterpart through the use of patch-clamp electrophysiology. With these experiments, we assessed whether this novel biosensor, bearing relatively large fusion proteins (i.e., GFP10 and RLucII), was a fully functional cationic channel. As TRPC7 is activated downstream of Gα_q_ signaling [5], we compared current–voltage profiles in HEK293 cells co-transfected with plasmids encoding Gα_q_-DREADD and either TRPC7 WT or GFP10-TRPC7-RLucII, as illustrated in Figure 2d. Following Gα_q_-DREADD stimulation with 1 µM of CNO, we observed no detectable difference between TRPC7 WT and GFP10-TRPC7-RLucII in the recorded currents.

Furthermore, we also assessed whether the change in BRET^2^ ratio was due to conformational changes of the TRPC7 channel in response to Gα_q_-DREADD activation or due to calcium entry through the channel pore. Our results showed that the normalized BRET^2^ ratios were similar between physiological buffer (containing calcium) and calcium-free buffer conditions (normalized Δ BRET^2^ ratio 0.118 ± 0.031 and 0.086 ± 0.021, respectively) (Figure S2a,b). Additionally, we evaluated the effect of SKF96365 (SKF), a non-selective calcium entry blocker, known to inhibit TRPC channels [24], on BRET^2^ ratio changes. Interestingly, after SKF treatment, a decrease in normalized BRET^2^ ratio (normalized Δ BRET^2^ ratio 0.087 ± 0.020 for CNO vs. control, and 0.068 ± 0.019 for CNO + SKF vs. SKF alone), similar to the non-treated condition, was observed (Figure S2c). Taken together, these results suggest that calcium influx does not hinder the function of the GFP10-TRPC7-RLucII biosensor.

Such tools have been reported by other groups to monitor TRP member conformational changes with TRPC3, shown to exhibit intramolecular changes using BRET^1^ [18]. This type of construct with BRET^1^ was also studied with another member of the TRP superfamily, TRPV1 (TRP Vanilloid 1) [19], recently demonstrating that this technology can be used for high-throughput screening [25]. This group also used NanoLuc tag, exhibiting less steric hindrance and higher signal, associated with the mNeonGreen tag to assess conformational changes of different TRP (TRPV4 and TRPM8) or non-TRP proteins (KCa2.3, Kir6.1, TREK1, rP2 × 2). Similar to the demonstrated functionality of the GFP10-TRPC7-RLucII biosensor, the intramolecular BRET-based TRPV1 biosensor was also assessed by patch-clamp [25]. This assay revealed that, despite the addition of two tags on the ion channel subunits, they remain functional. All of these results converge to confirm that BRET is a useful tool to study ion channel activation, especially for TRP members.

### 2.3. GFP10-TRPC7-RLucII Biosensor Competition with Untagged TRPC3 or TRPC7 Does Not Alter Its Conformational Changes in Response to Gα_q_-DREADD Stimulation

**Figure 3:**
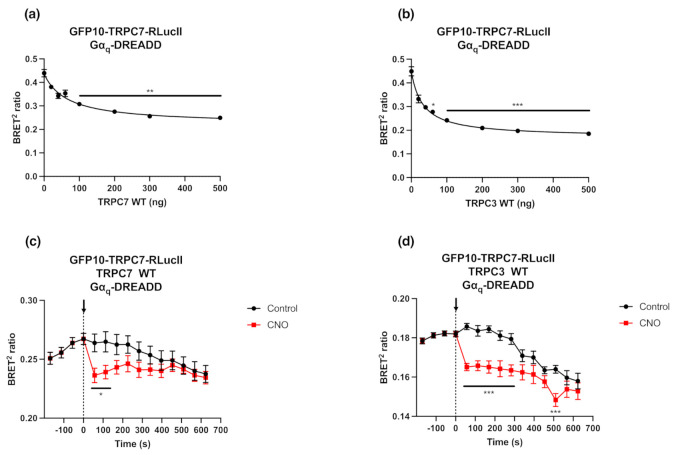
Double brilliance GFP10-TRPC7-RLucII biosensor also shows intermolecular interactions between TRPC7 subunits. HEK293 cells were co-transfected with plasmids encoding GFP10-TRPC7-RLucII (75 ng), Gα_q_-DREADD (500 ng) and increasing concentration of the corresponding untagged TRPC7 WT (**a**) or TRPC3 WT (**b**). Basal BRET ratio was measured. Statistical analyses were performed using a Kruskal–Wallis multiple comparisons followed by a Dunn’s post-hoc test. ** *p* < 0.01, *** *p* < 0.001 compared to no competition (0 ng of TRPC7 or TRPC3 WT). (**c**,**d**) HEK293 cells were co-transfected with plasmids encoding Gα_q_-DREADD (500 ng), GFP10-TRPC7-RLucII (75 ng), and TRPC7 WT (500 ng) (**c**) or TRPC3 WT (500 ng) (**d**) and were stimulated with CNO (1 µM) or vehicle as a control. BRET signal was measured for 10 min. Each data set represents the mean of three independent experiments, which were each performed in triplicate, and expressed as the mean ± S.E.M. Statistical analyses were performed using a two-way ANOVA with multiple comparisons followed by a Sidak’s post-hoc test. * *p* < 0.05, *** *p* < 0.001 for control vs. CNO.

Fully functional cationic TRPC channels are formed either as homotetramers or heterotetramers [26,27,28]. Thus, we overexpressed the untagged TRPC7 WT or one of its partners, TRPC3 WT, to verify whether the observed BRET signal changes were solely due to intramolecular changes in conformation of GFP10-TRPC7-RLucII or to the relative intermolecular changes upon pore opening in a homo- or hetero-tetrameric protein complex. The competition between GFP10-TRPC7-RLucII biosensor (75 ng) and increasing TRPC7 or TRPC3 WT (0 to 500 ng) showed a quantity-dependent and partial decrease in BRET ratio (Figure 3a,b). This pattern suggests that the BRET signal observed with the GFP10-TRPC7-RLucII biosensor was derived from both intra- and intermolecular interactions between the different subunits of the tetrameric protein complex. We then tested the response of this biosensor to Gα_q_-DREADD receptor’s activation with high levels of TRPC7 or TRPC3 WT expression (500 ng) (Figure 3c,d). Even if the Δ BRET^2^ ratios were lower in these conditions, the activation of the GFP10-TRPC7-RLucII biosensor remained detectable and significant, even in competition with untagged TRPC7 or TRPC3 WT after Gα_q_-DREADD stimulation.

BRET modulation in response to Gα_q_ activation, indicating conformational changes, was still detected in these experimental competition paradigms. The lower dynamic range following TRPC7 activation in competition experiments are still significant, compared to the previous experiments reported in Section 2.2 (0.028 ± 0.010 with TRPC7 WT and 0.021 ± 0.002 with TRPC3 WT vs. 0.052). The BRET signal monitored would be the result of not only intermolecular conformational changes within the heterotetrametric TRPC7 channel, but also integrated intramolecular conformational changes occurring within the single TRPC7 monomer. These experiments provide evidence that intermolecular interactions occur, although the intramolecular changes are readily detectable with a double-tagged ion channel biosensor. This could be extrapolated to other ion channel biosensors.

### 2.4. Gα_q_ Signaling Is Required to Induce Conformational Changes in GFP10-TRPC7-RLucII BRET-Based Biosensor

**Figure 4:**
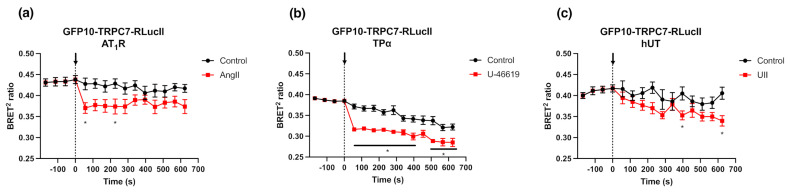
Gα_q_-coupled receptors modulate BRET ratio of GFP10-TRPC7-RLucII biosensor. (**a**–**c**) HEK293 cells co-transfected with plasmids encoding GFP10-TRPC7-RlucII biosensor (75 ng) and Gα_q_-coupled AT_1_R (**a**), TPα (**b**), or UT (**c**) receptors (500 ng) and were respectively stimulated with AngII, U-46619 and Urotensin (1 µM) or vehicle as a control. BRET signal was measured for 10 min. Each data set represents the mean of three independent experiments, which were each performed in triplicate, and expressed as the mean ± S.E.M. Statistical analyses were performed using a two-way ANOVA with multiple comparisons followed by a Sidak’s post-hoc test. * *p* < 0.05 for control vs. stimulation.

We next investigated the activation of GFP10-TRPC7-RLucII biosensor (75 ng) in response to different Gα_q_-coupled receptors (500 ng), namely the Angiotensin II type-1 receptor (AT_1_R), thromboxane A2 receptor alpha (TPα), and Urotensin II receptor (UT). The stimulation of these co-transfected receptors with saturating concentrations (1 µM) of Angiotensin II (AngII), U-46619 (a synthetic TPα agonist), and Urotensin II (UII), respectively, induced a decrease in BRET ratio (Figure 4a–c). Interestingly, the amplitude and kinetic of activation of the GFP10-TRPC7-RLucII biosensor under these conditions differed amongst the three tested Gα_q_-coupled receptors, suggesting divergent pharmacokinetic properties and/or relative propensity to activate Gα_q_ protein by their respective agonists.

**Figure 5:**
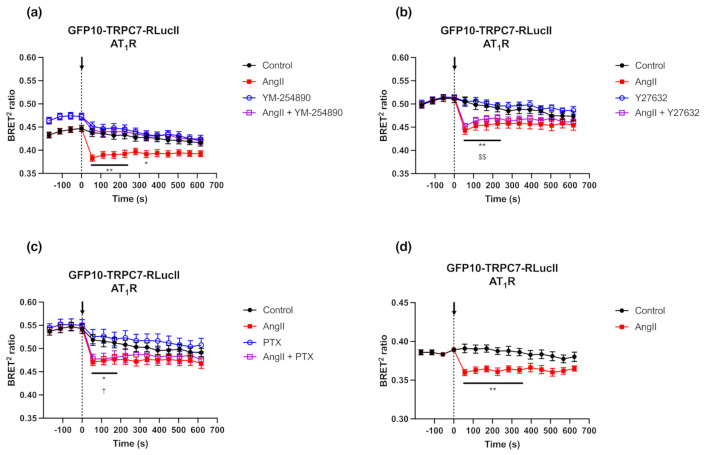
Modulation of BRET ratio in response to inhibition of specific G-protein signaling and loss of β-arrestins. (**a**–**c**) HEK293 cells were co-transfected with plasmids encoding AT_1_R (500 ng) and GFP10-TRPC7-RLucII (75 ng). Cells were pre-incubated 10 min with YM-254890 (Gαq inhibitor; 1 µM) (**a**), Y27632 (ROCK inhibitor; 10 µM) (**b**) or 24 h with Pertussis Toxin (PTX, Gαi/o inhibitor; 100 ng/mL) (**c**) before BRET measurement and stimulation with AngII (1 µM) or vehicle as a control. BRET signal was measured for 10 min. (**d**) βArr KO HEK293 cells were co-transfected with plasmids encoding AT_1_R (500 ng) and GFP10-TRPC7-RLucII (75 ng) and were respectively stimulated with AngII (1 µM) or vehicle as control. BRET signal was measured for 10 min. Each data set represents the mean of three independent experiments, which were each performed in triplicate, and expressed as the mean ± S.E.M. Statistical analyses were performed using a two-way ANOVA with multiple comparisons, followed by a Sidak’s post-hoc test. * *p* < 0.05, ** *p* < 0.01 for control vs. AngII; $$ *p* < 0.01 for Y27632 vs. AngII + Y27632; and † *p* < 0.05 for PTX vs. AngII + PTX.

Since GFP10-TRPC7-RLucII displayed a superior dynamic range upon AT_1_R agonist stimulation (0.057 ± 0.019), which has already been demonstrated to play a role in TRPC7 activation [14], and as compared to TPα (0.055 ± 0.007) and UT (0.022 ± 0.024), we further characterized this biosensor using AT_1_R, which is known to couple to Gα_q_, Gα_i/o_ and Gα_12/13_ [29,30,31]. Therefore, we investigated the effect of pharmacological inhibition of specific AT_1_R pathways by using the Gα_q/11_ inhibitor YM-254890 (1 µM), the Gα_i/o_ inhibitor pertussis toxin (PTX) (100 ng/mL), and the Rho kinase inhibitor Y27632 (10 µM), a downstream effector of the Gα_12/13_ pathway (Figure 5a–c). The activation of GFP10-TRPC7-RLucII was completely suppressed with YM-254890 (Figure 5a). In contrast, PTX and Y27632 did not impact the BRET signal of GFP10-TRPC7-RLucII activation (Figure 5b,c). Other members of the TRPC family, namely TRPC3 and TRPC5, have been demonstrated to be activated downstream of Gα_q/11_ and Gα_i/o_ [32,33,34]. Under our experimental conditions, the results demonstrate a predominant role of the Gα_q/11_ pathway in the activation of TRPC7 following AT_1_R stimulation.

Since the GPCR adapter protein β-arrestin 1 (βarr1) was found to be essential in TRPC3 activation following the binding of AngII to AT_1_R [18], we then assessed whether βarrs also affect TRPC7 activation. To this end, we co-transfected AT_1_R with the GFP10-TRPC7-RLucII biosensor in βArr KO HEK293 cells. Albeit the Δ BRET^2^ ratio is diminished in βArr KO HEK293 cells (0.031 ± 0.007), as compared to WT HEK293 cells (0.052 ± 0.014), AT_1_R stimulation by AngII still led to a decrease in the BRET^2^ ratio in βArr KO cells (Figure 5d), suggesting that βarrs are not crucial for TRPC7 activation. This differs from TRPC3, which depends on βArr1 to be activated [18]. Our results suggest that TRPC7 activation by GPCRs do not form a supramolecular complex at the cell membrane with the receptor and βarrs, as shown for TRPC3. We can assume that the decreased ΔBRET^2^ ratio is a consequence of the adaptation of HEK293 cells to CRISPR/Cas9 deletion of βArrs, certainly involving cell signaling rearrangement, and that the cellular context probably plays an important role in the activation of all TRPCs.

### 2.5. GFP10-TRPC7-RLucII Biosensor Shows Conformational Changes in Rat Cardiac Fibloblasts Expressing Endogenous AT_1_R

**Figure 6:**
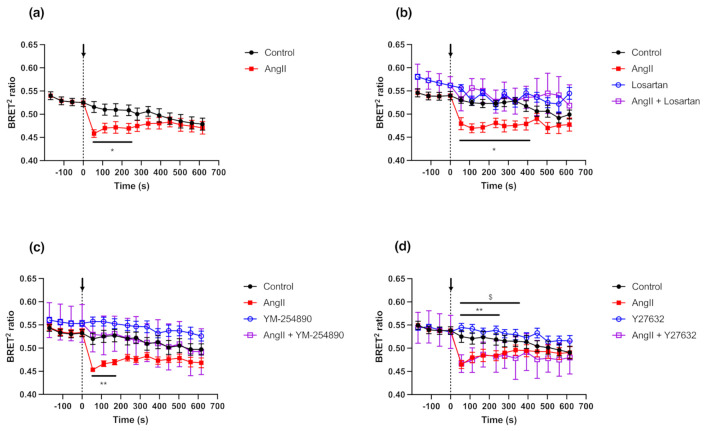
Stimulation of endogenous AT_1_R induces GFP10-TRPC7-RLucII biosensor conformational changes. Isolated rat neonatal cardiac fibroblasts were infected with the adenovirus encoding GFP10-TRPC7-RlucII biosensor. (**a**) Cells were stimulated with AngII (1 µM) 48 h post-infection, and BRET signal was measured for 10 min. Cells were pre-incubated with Losartan (100 µM) (**b**), YM-254890 (1 µM) (**c**) or Y27632 (10 µM) (**d**) before BRET measurement, and stimulation with AngII (1 µM) or vehicle as a control. BRET signal was measured for approximatively 10 min. Each data set represents the mean of three independent experiments, which were each performed in triplicate, and expressed as the mean ± S.E.M. Statistical analyses were performed using a two-way ANOVA with multiple comparisons, followed by a Sidak’s post-hoc test. * *p* < 0.05, ** *p* < 0.01 for control vs. AngII and $ *p* < 0.05 for Y27632 vs. AngII + Y27632.

TRPC7 and AT_1_R have been shown to be expressed in rat cardiac fibroblasts [35,36,37]. Satoh et al. (2007) have linked AT_1_R stimulation and TRPC7 activation in rat cardiomyocytes, and our results showed that TRPC7 is activated downstream of AT_1_R in HEK293 cells. Therefore, we assessed whether AT_1_R endogenously expressed in primary cell culture of rat neonatal cardiac fibroblasts (RNCFs) can also modulate the BRET responses of the GFP10-TRPC7-RLucII biosensor. RNCFs were isolated and infected with an adenovirus encoding GFP10-TRPC7-RLucII biosensor 4 h after plating, and the BRET signals were measured 48 h post-infection (Figure 6a). Following stimulation with AngII (1 µM), a decrease of the BRET^2^ ratio was observed in RNCFs.

Since both AT_1_R and AT_2_R subtypes are expressed in cardiac fibroblasts [37,38,39], we assessed whether the activation of TRPC7 BRET biosensor is mainly due to AT_1_R stimulation and not AT_2_R using Losartan, a selective AT_1_R antagonist (Figure 6b). Pre-incubation of Losartan with RNCFs completely abolished the activation of the GFP10-TRPC7-RLucII biosensor following AngII stimulation. We also confirmed the implication of Gα_q_ signaling pathway in mediating TRPC7 activation after AT_1_R stimulation by using YM-254890 and Y27632 in these primary cultured cells (Figure 6c,d). YM-254890 completely prevented the GFP10-TRPC7-RLucII biosensor activation (Figure 6c), whereas Y27632 did not affect its activation following AngII stimulation (Figure 6d). These results are in accordance with the findings in HEK293 cells as shown in Section 2.4.

Overall, these results confirmed that TRPC7 activation in NRCFs can be efficiently triggered by AT_1_R stimulation under physiological conditions. Our results are in line with previous studies highlighting the presence and function of TRPC7 in cardiac fibroblasts [35,36]. Moreover, TRPC7 and other TRPC are recognized to be important contributors, together with the canonical Transforming Growth Factor β (TGFβ) and AT_1_R pathways [40,41], in the development of cardiac fibrosis, and to exert essential roles in cardiac physiological and pathophysiological processes [42,43]. Considering the major role of Ca^2+^ signaling in the pathophysiological processes leading to cardiac fibrosis [44], the use of this novel GFP10-TRPC7-RLucII BRET biosensor may therefore help to decipher new pathways contributing to fibrosis.

## 3. Materials and Methods

### 3.1. Materials

Angiotensin II, U-46619, Urotensin II, Y27632, and SKF96365 were acquired from Sigma-Aldrich (St. Louis, MO, USA), Clozapine-N-Oxide (CNO) and Losartan were purchased from Tocris (Toronto, ON, Canada), YM-254890 was purchased from Adipogen Life Sciences Inc (San Diego, CA, USA), and Pertussis Toxin (PTX) was acquired from List Biological Laboratories (Campbell, CA, USA). Dulbecco’s modified Eagle’s medium (DMEM), fetal bovine serum (FBS), penicillin-streptomycin-glutamine (PSG), Hank’s balance salt solution (HBSS), and (4-(2-hydroxyethyl)-1-piperazineethanesulfonic acid) (HEPES) were purchased from Wisent (St-Bruno, QC, Canada). TryPLE Express Enzyme with phenol red was obtained from Gibco (Gaithersburg, MD, USA). Opti-MEM was purchased from Invitrogen (Burlington, ON, Canada). Polyethylenimine (PEI) was acquired from Polyscience (Warrington, PA, USA). Coelenterazine 400 A was from GoldBio (St. Louis, MO, USA). YM-254890 was used as described in other studies [45,46], i.e., preincubated 10 min at 1 µM. Other inhibitors were used as in our previous study [47]: Y27632 at 10 µM and Losartan at 100 µM (one hundred times AngII concentration) were preincubated 10 min before BRET reading, whereas PTX was preincubated at 100 ng/mL overnight.

### 3.2. Plasmids

Plasmid encoding the human Angiotensin II type-1 receptor (AT_1_R) was kindly provided by Dr. Sylvain Meloche (Université de Montréal, Montréal, QC, Canada). The plasmid coding for the SNAP-tagged urotensin II receptor (UT) was obtained from Cisbio Bioassays (Codolet, France), and the one containing Thromboxane A2 receptor alpha (TPα) was from cDNA.org. Gα_q_-DREADD was acquired from Addgene (cat#45547) (Watertown, MA, USA). The empty plasmids pcDNA3.1zeocin-RLucII/GFP10 and pcDNA3.1zeocin-GFP10/RLucII were a gift from Dr. Michel Bouvier (Université de Montréal, Montréal, QC, Canada). β-arrestin 1-Flag and β-arrestin 2-Flag plasmids were kindly given by Dr. Stéphane A. Laporte (McGill University, Montréal, QC, Canada).

TRPC7 plasmid, in pLX304 vector, was obtained through DNASU plasmid repository (clone#HsCD00437023, Arizona State University, Tempe, AZ, USA). RLucII-TRPC7-GFP10 and GFP10-TRPC7-RLuc double brilliance BRET-based biosensors were generated by PCR amplification of the human TRPC7 cDNA. Primers used to amplify TRPC7 contained 15 bp from the pcDNA3.1(+) RLucII-GFP10 or pcDNA3.1(+) GFP10-RLucII vector to be subcloned using the Gibson HiFi Assembly (New England Biolabs, Whitby, ON, Canada), according to the manufacturer instructions. A list of the PCR primers used to amplify the TRPC7 moiety is reported in Table S1. All generated constructs were purified by Midi DNA preparation (Qiagen, Toronto, ON, Canada), and analyzed through Sanger sequencing and subsequent DNA alignment. Sequences of generated biosensors plasmids are provided as Sequences S1 and S2.

### 3.3. Cell Culture

Human embryonic kidney 293 (HEK293)-A cells were purchased from Life Technologies (Carlsbad, CA, USA). The SL-CRISPR βArr1/2 KO HEK293 cell lines (βArr KO HEK293) were generated by Prof. Stephane A. Laporte (McGill University, Montréal, QC, Canada) by using the CRISPR/Cas9 method to delete β-arrestin 1 and β-arrestin 2, from a subclone of HEK293-A cells (HEK293-SL used as a control in β-arrestin experiments) [48]. Cells were maintained in DMEM medium supplemented with 10% FBS, penicillin (100 U/mL)-streptomycin (100 μg/mL)-glutamine (2 mM) at 37 °C in a humidified 5% CO_2_ atmosphere.

### 3.4. BRET Assays

To monitor TRPC7 activation, we used plasmids encoding intramolecular biosensors (i.e., RLucII-TRPC-GFP10 and GFP10-TRPC7-RLucII) or TRPC7 WT, co-transfected with plasmids containing cDNAs encoding different receptors. pIRES(puro)-RLucII vector was used to calculate the netBRET ratio of intramolecular biosensor activation subtracting the BRET background. Cells were transiently transfected with the indicated constructs at a density of 3.5 × 10^5^ cells/mL using a 3:1 ratio of linear PEI (1 mg/mL) per µg DNA [49]. A maximum of 2 µg of plasmid DNA was prepared in Opti-MEM to transfect 3.5 × 10^5^ cells. Salmon sperm DNA was used to keep the same amount of DNA under each condition. Cells were then directly seeded (35 × 10^5^ cells/well, 100 µL) in white opaque 96-well plates (BD Falcon, Corning, NY, USA).

After 48 h post-transfection, the medium was removed and replaced by HBSS containing 20 mM of HEPES, and left 1 h at 37 °C. The luciferase substrate coelenterazine 400 A (5 µM) was added 5 min prior to reading the BRET signal, and followed by the addition of ligands. Inhibitors were added 10 min before BRET reading, except for the PTX, which was added 24 h prior to the BRET reading. RLucII and GFP10 signals were acquired using a Berthold TriStar2 LB 942 Multimode Reader (Berthold, Bad Wildbad, Germany) equipped with high-sensitivity BRET^2^ filters (RLucII emission 410 nm/GFP10 emission 515 nm). BRET^2^ ratio was determined by dividing acceptor signal (GFP10) over donor signal (RLucII).

### 3.5. Electrophysiological Measurements

To monitor the electrophysiological activity, the maintenance and transfection of HEK293 cells were performed as previously described [50]. HEK293 cells were grown on glass coverslips in a 60 mm dish, were transfected with cDNAs encoding TRPC7 WT (0.7 µg) or GFP10-TRPC7-RLucII (0.7 µg), Gαq-DREADD receptor (1 µg), and Green Fluorescent Protein (GFP) (0.3 µg), and used within 48 h.

The methods for electrophysiology were previously described [51]. Briefly, cells were placed into a 2 mL bath solution containing (in mM): 140 NaCl, 1.5 CaCl_2_, 2 MgCl_2_, 5 KCl, 10 HEPES, 10 D-glucose (pH 7.4 adjusted with NaOH). Borosilicate glass (Harvard Apparatus Ltd., Holliston, MA, USA) pipettes were pulled and polished to 2–5 MΩ resistance with a DMZ-Universal Puller (Zeitz-Instruments GmbH, Martinsried, Germany), and filled with an internal solution containing (in mM): 120 CsCl, 10 EGTA, 10 HEPES, 3 MgCl_2_, 2 ATP, and 0.5 GTP, pH 7.2 adjusted with CsOH. Recordings were performed using an Axopatch 200 B amplifier (Axon Instruments, Hawthorn East, Australia). Cells were voltage-clamped, and TRP currents were measured using conventional whole-cell patch-clamp method. All the recordings were performed at room temperature (22 ± 2 °C). Currents were elicited by a ramp protocol from -100 mV to +100 mV at 10 s interval. CNO (1 µM), dissolved in the bath solution, was delivered using a gravitational perfusion system (ALA-VM8, Scientific Instruments, Columbia, MD, USA) at a rate of 1–2 mL/min. Voltage clamp protocols were applied using pClamp 10.4 software (Axon Instruments). Data were filtered at 1 kHz (8-pole Bessel) and digitized at 10 kHz with a Digidata 1440 A converter (Axon Instruments). For whole-cell recordings, the series resistance was compensated.

### 3.6. Cardiac Fibroblast Isolation and Infection

TRPC7 WT and GFP10-TRPC7-RLucII were subcloned into the Gateway pENTR3C Dual Selection vector (Invitrogen) using the NEBuilder HiFi DNA Assembly Cloning Kit (New England Biolabs). pENTR3C Dual Selection vectors were recombined in the Gateway pAd/CMV/V5-DEST adenovirus vector (Invitrogen) using the Gateway LR Clonase II Enzyme Mix (Invitrogen). The plasmids (4.5 μg) were digested with *Pac*I (New England Biolabs), and transfected into 80% confluent HEK293A cells cultured in 35-mm dishes with Lipofectamine 2000 transfection reagent (Invitrogen). The primary adenoviruses were collected seven days after the transfection from the medium, and amplified with 10 cm dishes of 90–100% confluent HEK293A cells. To determine the titer of newly generated adenoviruses, HEK293A cells were infected with serial dilutions of the viral particles, fixed with 100% methanol 36 h post-infection, stained with a specific anti-hexon antibody (Abcam, Cambridge, UK) and a secondary anti-mouse Alexa 488 antibody (Cell Signaling, Danvers, MA, USA), and imaged with the Operetta System (Perkin Elmer, Waltham, MA, USA) at 10× magnification.

Neonatal rat cardiac fibroblasts (NRCFs) and ventricular myocytes (NRVMs) were extracted from the hearts of 1–3 days-old Sprague–Dawley rat pups (Charles River Laboratories), as previously described [52]. Briefly, after trypsin and collagenase digestion, NRCFs and NRVMs were separated by a pre-plating step of 30 min at 37 °C, where NRCFs attached more rapidly than NRVMs to the petri dish. The supernatant containing NRVMs was resuspended in M199 medium supplemented with 10% FBS, penicillin (100 U/mL)-streptomycin (100 μg/mL)-glutamine (2 mM), and plated on gelatin-coated 96-well plates at a density of 10 × 10^5^ cells per well. NRCFs were cultured in DMEM supplemented with 10% FBS, penicillin (100 U/mL)-streptomycin (100 μg/mL)-glutamine (2 mM) and used at passage 3, at which point they were plated in 96-well plates at a density of 10 × 10^5^ cells per well. Four hours after plating, NRCFs and NRVMs were infected with adenoviruses encoding GFP10-TRPC7-RLucII at a multiplicity of infection of 50, and then cultured for 48 h before BRET assay.

### 3.7. Data Analysis

For BRET assays, raw data were imported in GraphPad Prism 9 software (San Diego, CA, USA), and each result is represented as mean ± S.E.M. of at least three independent experiments performed in triplicate. netBRET was calculated by subtracting background luminescence of cells only expressing *Renilla* Luciferase.

For electrophysiological assay, data analysis and offline leak subtraction were completed in Clampfit 10.4 (Axon Instruments), and all the analysis was performed using Origin 7.0 analysis software (OriginLab, Northampton, MA, USA).

Statistical analyses were performed using GraphPad Prism 9 and are described in the figure legend when applicable. A value was considered statistically significant when *p* < 0.05.

## 4. Conclusions

In this study, we report the construction of new tools to monitor conformational changes of TRPC7, an ion channel particularly involved in Ca^2+^ homeostasis. Using BRET-based constructions of TRPC7, we were able to record TRPC7 conformational changes in living cells with a fully functional channel, and to link the changes of conformation of TRPC7 to its activation. Using adenoviruses to package the TRPC7 biosensor, we effectively transduced and monitored TRPC7 activation in primary cells (cardiac fibroblasts). These new tools can therefore be used to decipher mechanistic events leading to TRPC7 activation in various cellular and pathophysiological contexts. Furthermore, BRET is a highly sensitive and robust technique that is suitable for high throughput screening, thus these biosensors can be used in the discovery of new TRPC7 modulators. The biosensor design described in this study can be applied not only to other members of the TRPC family, but also to the broader TRP channel family.

## Data Availability

The data presented in this study are available in the article and the supplementary information files.

## Supplementary Materials

The following supporting information can be downloaded at: https://www.mdpi.com/article/10.3390/ijms23052502/s1.

## References

[1] Vannier B., Peyton M., Boulay G., Brown D., Qin N., Jiang M., Zhu X., Birnbaumer L. (1999). Mouse trp2, the homologue of the human trpc2 pseudogene, encodes mTrp2, a store depletion-activated capacitative Ca^2+^ entry channel. Proc. Natl. Acad. Sci. USA.

[2] Vazquez G., Wedel B.J., Aziz O., Trebak M., Putney J.W. (2004). The mammalian TRPC cation channels. Biochim. Biophys. Acta.

[3] Monteith G.R., Prevarskaya N., Roberts-Thomson S.J. (2017). The calcium–cancer signalling nexus. Nat. Rev. Cancer.

[4] Hofmann T., Obukhov A.G., Schaefer M., Harteneck C., Gudermann T., Schultz G. (1999). Direct activation of human TRPC6 and TRPC3 channels by diacylglycerol. Nature.

[5] Okada T., Inoue R., Yamazaki K., Maeda A., Kurosaki T., Yamakuni T., Tanaka I., Shimizu S., Ikenaka K., Imoto K. (1999). Molecular and Functional Characterization of a Novel Mouse Transient Receptor Potential Protein Homologue TRP7. J. Biol. Chem..

[6] Numaga T., Wakamori M., Mori Y., Flockerzi V., Nilius B. (2007). TRPC7. Transient Receptor Potential (TRP) Channels.

[7] Hsu W., Tsai M., Wu C., Liang J., Lu J., Kahle J.S., Yu H., Yen C., Yen C., Hsieh Y. (2019). Nociceptive transient receptor potential canonical 7 (TRPC7) mediates aging-associated tumorigenesis induced by ultraviolet B. Aging Cell.

[8] Beck B., Zholos A., Sydorenko V., Roudbaraki M., Lehen’Kyi V., Bordat P., Prevarskaya N., Skryma R. (2006). TRPC7 Is a Receptor-Operated DAG-Activated Channel in Human Keratinocytes. J. Investig. Dermatol..

[9] Lievremont J.-P., Numaga T., Vazquez G., Lemonnier L., Hara Y., Mori E., Trebak M., Moss S.E., Bird G.S., Mori Y. (2005). The Role of Canonical Transient Receptor Potential 7 in B-cell Receptor-activated Channels. J. Biol. Chem..

[10] Itsuki K., Imai Y., Hase H., Okamura Y., Inoue R., Mori M.X. (2014). PLC-mediated PI(4,5)P2 hydrolysis regulates activation and inactivation of TRPC6/7 channels. J. Gen. Physiol..

[11] Vazquez G., Bird G.S.J., Mori Y., Putney J. (2006). Native TRPC7 Channel Activation by an Inositol Trisphosphate Receptor-dependent Mechanism. J. Biol. Chem..

[12] Lemonnier L., Trebak M., Putney J.W. (2008). Complex regulation of the TRPC3, 6 and 7 channel subfamily by diacylglycerol and phosphatidylinositol-4,5-bisphosphate. Cell Calcium.

[13] Phelan K.D., Shwe U.T., Abramowitz J., Birnbaumer L., Zheng F. (2014). Critical role of canonical transient receptor potential channel 7 in initiation of seizures. Proc. Natl. Acad. Sci. USA.

[14] Satoh S., Tanaka H., Ueda Y., Oyama J.-I., Sugano M., Sumimoto H., Mori Y., Makino N. (2006). Transient receptor potential (TRP) protein 7 acts as a G protein-activated Ca^2+^ channel mediating angiotensin II-induced myocardial apoptosis. Mol. Cell. Biochem..

[15] Gogebakan B., Bayraktar R., Suner A., Balakan O., Ulasli M., Izmirli M., Oztuzcu S., Camci C. (2014). Do Fasudil and Y-27632 affect the level of transient receptor potential (TRP) gene expressions in breast cancer cell lines?. Tumor Biol..

[16] Yamaguchi Y., Iribe G., Nishida M., Naruse K. (2017). Role of TRPC3 and TRPC6 channels in the myocardial response to stretch: Linking physiology and pathophysiology. Prog. Biophys. Mol. Biol..

[17] Onohara N., Nishida M., Inoue R., Kobayashi H., Sumimoto H., Sato Y., Mori Y., Nagao T., Kurose H. (2006). TRPC3 and TRPC6 are essential for angiotensin II-induced cardiac hypertrophy. EMBO J..

[18] Liu C.-H., Gong Z., Liang Z.-L., Liu Z.-X., Yang F., Sun Y.-J., Ma M.-L., Wang Y.-J., Ji C.-R., Wang Y.-H. (2017). Arrestin-biased AT1R agonism induces acute catecholamine secretion through TRPC3 coupling. Nat. Commun..

[19] Ruigrok H.J., Shahid G., Goudeau B., de Gannes F.P., Poque-Haro E., Hurtier A., Lagroye I., Vacher P., Arbault S., Sojic N. (2017). Full-Spectral Multiplexing of Bioluminescence Resonance Energy Transfer in Three TRPV Channels. Biophys. J..

[20] Charest P.G., Terrillon S., Bouvier M. (2005). Monitoring agonist-promoted conformational changes of β-arrestin in living cells by intramolecular BRET. EMBO Rep..

[21] Bacart J., Corbel C., Jockers R., Bach S., Couturier C. (2008). The BRET technology and its application to screening assays. Biotechnol. J..

[22] Hu J., Stern M., Gimenez L.E.D., Wanka L., Zhu L., Rossi M., Meister J., Inoue A., Beck-Sickinger A.G., Gurevich V.V. (2016). A G Protein-biased Designer G Protein-coupled Receptor Useful for Studying the Physiological Relevance of Gq/11-dependent Signaling Pathways. J. Biol. Chem..

[23] Takasaki J., Saito T., Taniguchi M., Kawasaki T., Moritani Y., Hayashi K., Kobori M. (2004). A Novel Gαq/11-selective Inhibitor. J. Biol. Chem..

[24] He X., Li S., Liu B., Susperreguy S., Formoso K., Yao J., Kang J., Shi A., Birnbaumer L., Liao Y. (2017). Major contribution of the 3/6/7 class of TRPC channels to myocardial ischemia/reperfusion and cellular hypoxia/reoxygenation injuries. Proc. Natl. Acad. Sci. USA.

[25] Chappe Y., Michel P., Joushomme A., Barbeau S., Pierredon S., Baron L., Garenne A., De Gannes F.P., Hurtier A., Mayer S. (2021). High-Throughput Screening of TRPV1 Ligands in the Light of the Bioluminescence Resonance Energy Transfer Technique. Mol. Pharmacol..

[26] Hofmann T., Schaefer M., Schultz G., Gudermann T. (2002). Subunit composition of mammalian transient receptor potential channels in living cells. Proc. Natl. Acad. Sci. USA.

[27] Venkatachalam K., Montell C. (2007). TRP Channels. Annu. Rev. Biochem..

[28] Myeong J., Ko J., Hong C., Yang D., Lee K.P., Jeon J.-H., So I. (2016). The interaction domains of transient receptor potential canonical (TRPC)1/4 and TRPC1/5 heteromultimeric channels. Biochem. Biophys. Res. Commun..

[29] St-Pierre D., Cabana J., Holleran B.J., Besserer-Offroy A., Escher E., Guillemette G., Lavigne P., Leduc R. (2018). Angiotensin II cyclic analogs as tools to investigate AT1R biased signaling mechanisms. Biochem. Pharmacol..

[30] Galandrin S., Denis C., Boularan C., Marie J., M’Kadmi C., Pilette C., Dubroca C., Nicaise Y., Seguelas M.-H., N’Guyen D. (2016). Cardioprotective Angiotensin-(1–7) Peptide Acts as a Natural-Biased Ligand at the Angiotensin II Type 1 Receptor. Hypertension.

[31] Namkung Y., LeGouill C., Kumar S., Cao Y., Teixeira L.B., Lukasheva V., Giubilaro J., Simões S.C., Longpré J.-M., Devost D. (2018). Functional selectivity profiling of the angiotensin II type 1 receptor using pathway-wide BRET signaling sensors. Sci. Signal..

[32] Jeon J.-P., Lee K.P., Park E.J., Sung T.S., Kim B.J., Jeon J.-H., So I. (2008). The specific activation of TRPC4 by Gi protein subtype. Biochem. Biophys. Res. Commun..

[33] Jeon J.-P., Hong C., Park E.-J., Jeon J.-H., Cho N.-H., Kim I.-G., Choe H., Muallem S., Kim H.J., So I. (2012). Selective Gα_i_ Subunits as Novel Direct Activators of Transient Receptor Potential Canonical (TRPC)4 and TRPC5 Channels. J. Biol. Chem..

[34] Zholos A.V. (2014). TRPC5. Mammalian Transient Receptor Potential (TRP) Cation Channels.

[35] Nishida M., Onohara N., Sato Y., Suda R., Ogushi M., Tanabe S., Inoue R., Mori Y., Kurose H. (2007). Gα12/13-mediated Up-regulation of TRPC6 Negatively Regulates Endothelin-1-induced Cardiac Myofibroblast Formation and Collagen Synthesis through Nuclear Factor of Activated T Cells Activation. J. Biol. Chem..

[36] Rose R.A., Hatano N., Ohya S., Imaizumi Y., Giles W.R. (2007). C-type natriuretic peptide activates a non-selective cation current in acutely isolated rat cardiac fibroblasts via natriuretic peptide C receptor-mediated signalling. J. Physiol..

[37] Villarreal F.J., Kim N.N., Ungab G.D., Printz M.P., Dillmann W.H. (1993). Identification of functional angiotensin II receptors on rat cardiac fibroblasts. Circulation.

[38] Van Kesteren C., Van Heugten H., Lamers J., Saxena P., Schalekamp M., Danser A. (1997). Angiotensin II-mediated Growth and Antigrowth Effects in Cultured Neonatal Rat Cardiac Myocytes and Fibroblasts. J. Mol. Cell. Cardiol..

[39] Dostal D.E., Rothblum K.N., Conrad K.M., Cooper G.R., Baker K.M. (1992). Detection of angiotensin I and II in cultured rat cardiac myocytes and fibroblasts. Am. J. Physiol. Physiol..

[40] Davis J., Burr A.R., Davis G.F., Birnbaumer L., Molkentin J.D. (2012). A TRPC6-Dependent Pathway for Myofibroblast Transdifferentiation and Wound Healing In Vivo. Dev. Cell.

[41] Davis J., Molkentin J.D. (2013). Myofibroblasts: Trust your heart and let fate decide. J. Mol. Cell. Cardiol..

[42] Londoño J.E.C., Marx A., Kraft A.E., Schürger A., Richter C., Dietrich A., Lipp P., Birnbaumer L., Freichel M. (2020). Angiotensin-II-Evoked Ca^2+^ Entry in Murine Cardiac Fibroblasts Does Not Depend on TRPC Channels. Cells.

[43] Wen H., Gwathmey J.K., Xie L.-H. (2020). Role of Transient Receptor Potential Canonical Channels in Heart Physiology and Pathophysiology. Front. Cardiovasc. Med..

[44] Feng J., Armillei M.K., Yu A.S., Liang B.T., Runnels L.W., Yue L. (2019). Ca^2+^ Signaling in Cardiac Fibroblasts and Fibrosis-Associated Heart Diseases. J. Cardiovasc. Dev. Dis..

[45] Besser L., Chorin E., Sekler I., Silverman W.F., Atkin S., Russell J.T., Hershfinkel M. (2009). Synaptically Released Zinc Triggers Metabotropic Signaling via a Zinc-Sensing Receptor in the Hippocampus. J. Neurosci..

[46] Zhang W., Sakoda H., Nakazato Y., Islam N., Pattou F., Kerr-Conte J., Nakazato M. (2021). Neuromedin U uses Gαi2 and Gαo to suppress glucose-stimulated Ca^2+^ signaling and insulin secretion in pancreatic β cells. PLoS ONE.

[47] Lavenus S., Simard É., Besserer-Offroy É., Froehlich U., Leduc R., Grandbois M. (2018). Label-free cell signaling pathway deconvolution of angiotensin type 1 receptor reveals time-resolved G-protein activity and distinct AngII and AngIIIIV responses. Pharmacol. Res..

[48] Namkung Y., Le Gouill C., Lukashova V., Kobayashi H., Hogue M., Khoury E., Song M., Bouvier M., Laporte S.A. (2016). Monitoring G protein-coupled receptor and β-arrestin trafficking in live cells using enhanced bystander BRET. Nat. Commun..

[49] Ehrhardt C., Schmolke M., Matzke A., Knoblauch A., Will C., Wixler V., Ludwig S. (2006). Polyethylenimine, a cost-effective transfection reagent. Signal Transduct..

[50] Flynn R., Chapman K., Iftinca M., Aboushousha R., Varela D., Altier C. (2014). Targeting the Transient Receptor Potential Vanilloid Type 1 (TRPV1) Assembly Domain Attenuates Inflammation-Induced Hypersensitivity. J. Biol. Chem..

[51] Iftinca M., Flynn R., Basso L., Melo H., Aboushousha R., Taylor L., Altier C. (2016). The stress protein heat shock cognate 70 (Hsc70) inhibits the Transient Receptor Potential Vanilloid type 1 (TRPV1) channel. Mol. Pain.

[52] Malette J., Degrandmaison J., Giguère H., Berthiaume J., Frappier M., Parent J.-L., Auger-Messier M., Boulay G. (2019). MURC/CAVIN-4 facilitates store-operated calcium entry in neonatal cardiomyocytes. Biochim. Biophys. Acta.

