# Supplementary material for: Monitoring TRPC7 Conformational Changes by BRET Following GPCR Activation
Source: Int J Mol Sci. 2022 Feb 24;23(5):2502. doi: 10.3390/ijms23052502 (PMC8910688; doi:10.3390/ijms23052502)
Supplement: Supplementary file 1 [file ijms-23-02502-s001.zip › ijms-1476538-supplementary.pdf]

Supplementary Information

Monitoring TRPC7 Conformational Changes by BRET Following GPCR Activation

Cécile Pétigny [1,2,3], Audrey-Ann Dumont [2,3,4], Hugo Giguère [2,3,4], Audrey Collette [1,2,3], Brian J. Holleran [1,2,3], Mircea Iftinca [5], Christophe Altier [5], Élie Besserer-Offroy [6,7,8], Mannix Auger-Messier [2,3,4] and Richard Leduc [1,2,3,*]

1 Department of Pharmacology-Physiology, Faculty of Medicine and Health Sciences, Université de Sherbrooke, Sherbrooke, QC J1H 5N4, Canada; cecile.petigny@usherbrooke.ca (C.P.); audrey.collette@usherbrooke.ca (A.C.); brian.holleran@usherbrooke.ca (B.J.H.)
2 Institut de Pharmacologie de Sherbrooke, Université de Sherbrooke, Sherbrooke, QC J1H 5N4, Canada
3 Centre de recherche du Centre Hospitalier Universitaire de Sherbrooke, Sherbrooke, QC J1H 5N4, Canada
4 Department of Medicine, Division of Cardiology, Faculty of Medicine and Health Sciences, Université de Sherbrooke, Sherbrooke, QC J1H 5N4, Canada; audrey-ann.dumont@usherbrooke.ca (A.-A.D.); hugo.giguere@usherbrooke.ca (H.G.); mannix.auger-messier@usherbrooke.ca (M.A.-M.)
5 Department of Physiology and Pharmacology, Inflammation Research Network-Snyder Institute for Chronic Diseases and Alberta Children's Hospital Research Institute, University of Calgary, Calgary, AB T2N 1N4, Canada; miftinca@ucalgary.ca (M.I.); altier@ucalgary.ca (C.A.)
6 Department of Molecular and Medical Pharmacology, Ahmanson Translational Theranostics Division, David Geffen School of Medicine, University of California-Los Angeles, Los Angeles, CA 90095, USA; ebesserer@ucla.edu (É. B.-O.)
7 Jonsson Comprehensive Cancer Center, University of California, Los Angeles, CA 90095, USA
8 California NanoSystems Institute, Los Angeles, CA 90095, USA
* Correspondence: richard.leduc@usherbrooke.ca (R.L.)

Copyright: © 2022 by the authors. Open access publication under the terms and conditions of the Creative Commons Attribution (CC BY) license (http://creativecommons.org/licenses/by/4.0/).

Table of Contents

Table S1 .... **page S3**

Figure S1 .... **page S4**

Figure S2 .... **page S5**

Sequence S1 .... **page S6**

Sequence S2 .... **page S8**

Table S1: PCR primers used for TRPC7 amplification.

Biosensor	Primer	DNA sequence
**RLUCII-TRPC7-GFP10**	Forward	gagcagggatccgccggtaccATGTTGAGGAACAGCACC
	Reverse	tgctcaccatggtggcgggaagcttAATGTCTTTGCCCTTGTTC
**GFP10-TRPC7-RLUCII**	Forward	tacaagggatccgccggtaccATGTTGAGGAACAGCACC
	Reverse	tgctggtcatggtggcgggaagcttAATGTCTTTGCCCTTGTTC

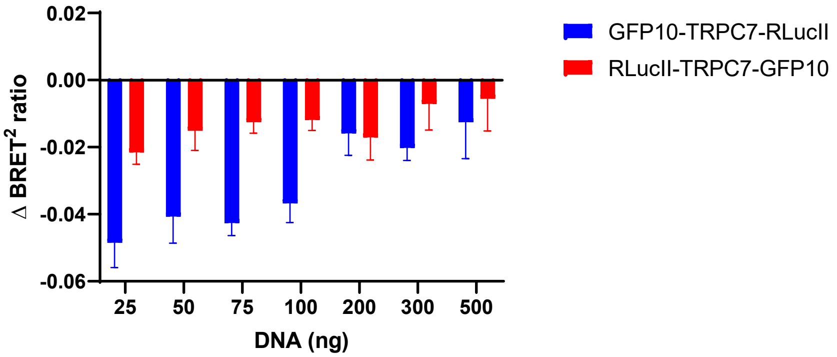

Figure S1: ΔBRET² ratio of double-tagged TRPC7 biosensors after Gα_q-DREADD activation by CNO. HEK293 cells were co-transfected with plasmids encoding RLucII-TRPC7-GFP10 or GFP10-TRPC7-RLucII biosensor and Gα_q-DREADD or co-transfected with an empty vector containing RLucII tag and Gα_q-DREADD. BRET signal was measured after stimulation with 1 μM of Clozapine-N-Oxide (CNO) or vehicle as a control. BRET was calculated by subtracting background luminescence of cells only expressing RLucII, approximately 60s after stimulation.

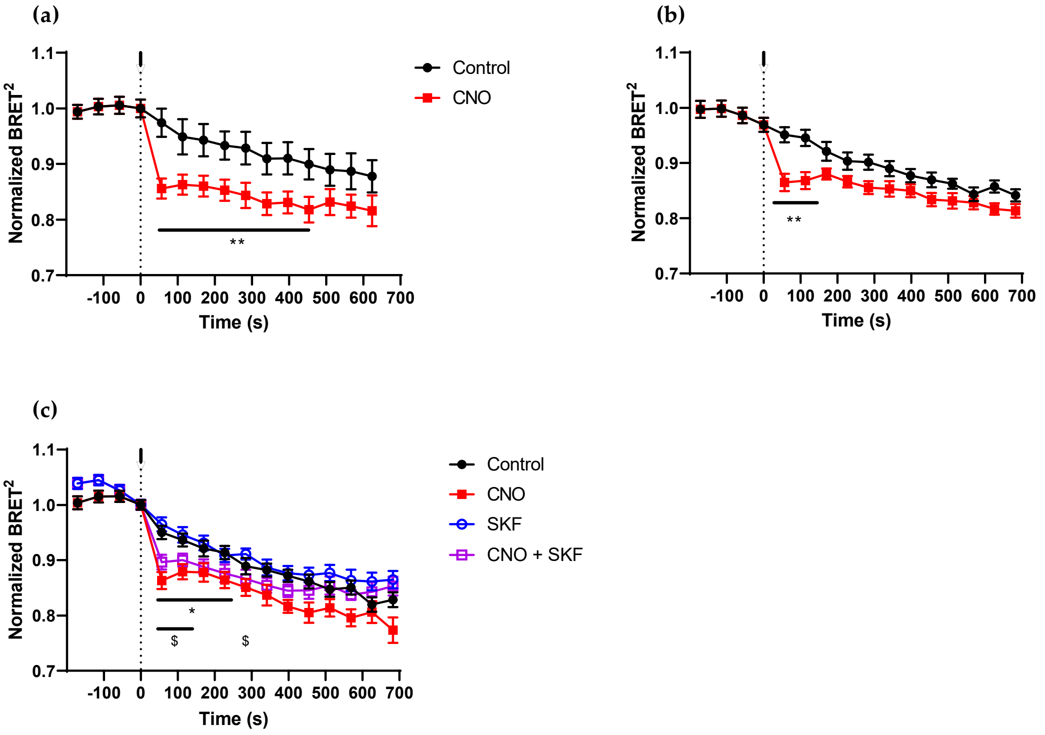

Figure S2: Modulation of BRET ratio in calcium-free conditions and response to pharmacological calcium entry blockade. HEK293 cells were co-transfected with plasmids encoding GFP10-TRPC7-RLucII (75 ng), Gαq-DREADD (500 ng) and were stimulated with CNO, 1 µM or vehicle as a control. The BRET signal was measured for 10 minutes in HBSS buffer containing calcium (a) or in calcium-free HBSS buffer replaced 5 min before acquisition (b). (c) HEK293 cells were transfected in the same conditions as in (a) and were pre-incubated 10 minutes with SKF96365 (SKF; 10 µM), a non-selective TRPC channel calcium entry blocker before BRET measurement and stimulation with CNO (1 µM) or vehicle as a control. BRET signal was measured for 10 minutes. Each data set represents the mean of three independent experiments, which were each done in triplicate, and expressed as the mean ± S.E.M. Statistical analyses were performed using a Two-Way ANOVA with multiple comparisons followed by a Sidak's post-hoc test. * $p < 0.05$, ** $p < 0.01$ for control *vs.* CNO; $ $p < 0.05$ for SKF *vs.* CNO + SKF.

Sequence S1: DNA sequence of GFP10-TRPC7-RlucII biosensor.

ATACCAGGCGTTTCCCCCTGGAAGCTCCCTCGTGCGCTCTCCTGTTCCGACCCTGCCGCTTACCGGATACCTGTCCGCCTTTCTCCCTT
CGGGAAGCGTGGCGCTTTCTCAATGCTCACGCTGTAGGTATCTCAGTTCGGTGTAGGTCGTTCGCTCCAAGCTGGGCTGTGTGCACGAA
CCCCCCGTTCAGCCCGACCGCTGCGCCTTATCCGGTAACTATCGTCTTGAGTCCAACCCGGTAAGACACGACTTATCGCCACTGGCAGC
AGCCACTGGTAACAGGATTAGCAGAGCGAGGTATGTAGGCGGTGCTACAGAGTTCTTGAAGTGGTGGCCTAACTACGGCTACACTAGAA
GGACAGTATTTGGTATCTGCGCTCTGCTGAAGCCAGTTACCTTCGGAAAAAGAGTTGGTAGCTCTTGATCCGGCAAACAAACCACCGCT
GGTAGCGGTGGTTTTTTTGTTTGCAAGCAGCAGATTACGCGCAGAAAAAAAGGATCTCAAGAAGATCCTTTGATCTTTTCTACGGGGTC
TGACGCTCAGTGGAACGAAAACTCACGTTAAGGGATTTTGGTCATGAGATTATCAAAAAGGATCTTCACCTAGATCCTTTTAAATTAAA
AATGAAGTTTTAAATCAATCTAAAGTATATATGAGTAAACTTGGTCTGACAGTTACCAATGCTTAATCAGTGAGGCACCTATCTCAGCG
ATCTGTCTATTTCGTTCATCCATAGTTGCCTGACTCCCCGTCGTGTAGATAACTACGATACGGGAGGGCTTACCATCTGGCCCCAGTGC
TGCAATGATACCGCGAGACCCACGCTCACCGGCTCCAGATTTATCAGCAATAAACCAGCCAGCCGGAAGGGCCGAGCGCAGAAGTGGTC
CTGCAACTTTATCCGCCTCCATCCAGTCTATTAATTGTTGCCGGGAAGCTAGAGTAAGTAGTTCGCCAGTTAATAGTTTGCGCAACGTT
GTTGCCATTGCTACAGGCATCGTGGTGTCACGCTCGTCGTTTGGTATGGCTTCATTCAGCTCCGGTTCCCAACGATCAAGGCGAGTTAC
ATGATCCCCCATGTTGTGCAAAAAAGCGGTTAGCTCCTTCGGTCCTCCGATCGTTGTCAGAAGTAAGTTGGCCGCAGTGTTATCACTCA
TGGTTATGGCAGCACTGCATAATTCTCTTACTGTCATGCCATCCGTAAGATGCTTTTCTGTGACTGGTGAGTACTCAACCAAGTCATTC
TGAGAATAGTGTATGCGGCGACCGAGTTGCTCTTGCCCGGCGTCAATACGGGATAATACCGCGCCACATAGCAGAACTTTAAAAGTGCT
CATCATTGGAAAACGTTCTTCGGGGCGAAAACTCTCAAGGATCTTACCGCTGTTGAGATCCAGTTCGATGTAACCCACTCGTGCACCCA
ACTGATCTTCAGCATCTTTTACTTTCACCAGCGTTTCTGGGTGAGCAAAAACAGGAAGGCAAAATGCCGCAAAAAAGGGAATAAGGGCG
ACACGGAAATGTTGAATACTCATACTCTTCCTTTTTCAATATTATTGAAGCATTTATCAGGGTTATTGTCTCATGAGCGGATACATATT
TGAATGTATTTAGAAAAATAAACAAATAGGGGTTCCGCGCACATTTCCCCGAAAAGTGCCACCTGACGTCGACGGATCGGGAGATCTCC
CGATCCCCTATGGTCGACTCTCAGTACAATCTGCTCTGATGCCGCATAGTTAAGCCAGTATCTGCTCCCTGCTTGTGTGTTGGAGGTCG
CTGAGTAGTGCGCGAGCAAAATTTAAGCTACAACAAGGCAAGGCTTGACCGACAATTGCATGAAGAATCTGCTTAGGGTTAGGCGTTTT
GCGCTGCTTCGCGATGTACGGGCCAGATATACGCGTTGACATTGATTATTGACTAGTTATTAATAGTAATCAATTACGGGGTCATTAGT
TCATAGCCCATATATGGAGTTCCGCGTTACATAACTTACGGTAAATGGCCCGCCTGGCTGACCGCCCAACGACCCCCGCCCATTGACGT
CAATAATGACGTATGTTCCCATAGTAACGCCAATAGGGACTTTCCATTGACGTCAATGGGTGGACTATTTACGGTAAACTGCCCACTTG
GCAGTACATCAAGTGTATCATATGCCAAGTACGCCCCCTATTGACGTCAATGACGGTAAATGGCCCGCCTGGCATTATGCCCAGTACAT
GACCTTATGGGACTTTCCTACTTGGCAGTACATCTACGTATTAGTCATCGCTATTACCATGGTGATGCGGTTTTGGCAGTACATCAATG
GGCGTGGATAGCGGTTTGACTCACGGGGATTTCCAAGTCTCCACCCCATTGACGTCAATGGGAGTTTGTTTTGGCACCAAAATCAACGG
GACTTTCCAAAATGTCGTAACAACTCCGCCCCATTGACGCAAATGGGCGGTAGGCGTGTACGGTGGGAGGTCTATATAAGCAGAGCTCT
CTGGCTAACTAGAGAACCCACTGCTTACTGGCTTATCGAAATTAATACGACTCACTATAGGGAGACCCAAGCTGGCTAGCCACCATGGT
GAGCAAGGGCGAGGAGCTGTTCACCGGGGTGGTGCCCATCCTGGTCGAGCTGGACGGCGACGTAAACGGCCACAAGTTCAGCGTGTCCG
GCGAGGGCGAGGGCGATGCCACCTACGGCAAGCTGACCCTGAAGTTCATCTGCACCACCGGCAAGCTGCCCGTGCCCTGGCCCACCCTC
GTGACCACCCTGAGCTACGGCGTGCAGTGCTTCAGCCGCTACCCCGACCACATGAAGCAGCACGACTTCTTCAAGTCCGCCATGCCCGA
AGGCTACGTCCAGGAGCGCACCATCTTCTTCAAGGACGACGGCAACTACAAGACCCGCGCCGAGGTGAAGTTCGAGGGCGACACCCTGG
TGAACCGCATCGAGCTGAAGGGCATCGACTTCAAGGAGGACGGCAACATCCTGGGGCACAAGCTGGAGTACAACTACAACCCCCACAAC
GTCTATATCATGGCCGACAAGCAGAAGAACGGCATCAAGGTGAACTTCAAGATCCGCCACAACATCGAGGACGGCAGCGTGCAGCTCGC
CGACCACTACCAGCAGAACACCCCCATCGGCGACGGCCCCGTGCTGCTGCCCGACAACCACTACCTGTTCACCCAGTCCGCCCTGAGCA
AAGACCCCAACGAGAAGCGCGATCACATGGTCCTGCTGGAGTTCGTGACCGCCGCCGGGATCACTCTCGGCATGGACGAGCTGTACAAG
GGATCCGCCGGTACCATGTTGAGGAACAGCACCTTCAAAAACATGCAGCGCCGGCACACAACGCTGAGGGAGAAGGGCCGTCGCCAGGC
CATCCGGGGTCCCGCCTACATGTTCAACGAGAAGGGCACCAGTCTGACGCCCGAGGAGGAGCGCTTCCTGGACTCGGCTGAGTATGGCA
ACATCCCGGTGGTCCGGAAAATGCTGGAGGAGTCCAAGACCCTTAACTTCAACTGTGTGGACTACATGGGGCAGAACGCTCTGCAGCTG
GCCGTGGGCAACGAGCACCTAGAGGTCACGGAGCTGCTGCTGAAGAAGGAGAACCTGGCACGGGTGGGGGACGCGCTGCTGCTGGCCAT
CAGCAAGGGCTATGTGCGCATCGTGGAGGCCATCCTCAACCACCCGGCCTTCGCGCAGGGCCAGCGCCTGACGCTCAGCCCGCTGGAAC
AGGAGCTGCGCGACGACGACTTCTATGCCTACGACGAGGACGGCACGCGCTTCTCCCACGACATCACGCCCATCATCCTGGCGGCGCAC
TGCCAGGAGTATGAGATCGTGCACATCCTGCTGCTCAAGGGCGCCCGCATCGAGCGGCCCCACGACTACTTCTGCAAGTGCAATGAGTG
CACCGAGAAACAGCGGAAAGACTCCTTCAGCCACTCGCGCTCGCGCATGAACGCCTACAAAGGACTGGCGAGTGCTGCCTACTTGTCCC
TGTCCAGCGAAGACCCTGTCCTCACCGCCCTGGAGCTCAGCAACGAGTTAGCCAGACTAGCCAACATTGAGACTGAATTTAAGAACGAT
TACAGGAAGTTATCTATGCAATGCAAGGATTTTGTAGTGGGCGTGCTGGACCTGTGCCGAGACACAGAAGAGGTGGAAGCAATTTTAAA
CGGTGATGTGAACTTCCAAGTCTGGTCCGACCACCACCGTCCAAGTCTGAGCCGGATCAAACTCGCCATTAAATATGAAGTCAAGAAGT
TCGTTGCTCATCCTAACTGTCAGCAGCAATTGCTTACCATGTGGTATGAAAATCTCTCAGGCTTACGTCAACAGTCTATCGCTGTGAAA
TTCCTGGCTGTCTTTGGAGTCTCCATAGGCCTCCCTTTTCTCGCCATAGCCTATTGGATTGCTCCGTGCAGCAAGCTAGGACGAACCCT
GAGGAGCCCTTTCATGAAGTTTGTAGCTCATGCAGTTTCTTTTACAATCTTCTTGGGATTATTAGTTGTGAATGCATCTGACCGATTTG
AAGGTGTTAAAACCCTGCCAAACGAAACCTTCACAGACTACCCAAAACAAATCTTCAGAGTGAAAACCACACAGTTCTCCTGGACAGAA
ATGCTCATTATGAAGTGGGTCTTAGGAATGATTTGGTCCGAATGCAAGGAAATCTGGGAGGAGGGGCCACGGGAGTACGTGCTGCACTT
GTGGAACCTGCTAGATTTCGGGATGCTGTCCATCTTCGTGGCCTCCTTCACAGCACGCTTCATGGCCTTCCTGAAGGCCACGGAGGCAC
AGCTGTACGTGGACCAGCACGTGCAGGACGACACGCTGCACAATGTCTCGCTTCCGCCGGAAGTGGCATACTTCACCTACGCCAGGGAC
AAGTGGTGGCCTTCAGACCCTCAGATCATATCGGAAGGGCTCTACGCGATAGCCGTCGTGCTGAGCTTCTCTCGCATTGCATACATTCT
GCCAGCCAACGAGAGTTTTGGGCCCCTGCAGATCTCGCTAGGGAGAACTGTGAAAGATATCTTCAAGTTCATGGTCATTTTCATCATGG
TATTTGTGGCCTTCATGATTGGGATGTTCAACCTGTACTCTTACTACCGAGGTGCCAAATACAACCCAGCGTTTACAACGGTTGAAGAA

AGTTTTAAAACTTTGTTTTGGTCCATATTCGGCTTATCTGAAGTAATCTCAGTGGTGCTGAAATACGACCACAAATTCATCGAGAACAT
TGGCTACGTTCTCTACGGCGTTTATAACGTCACCATGGTGGTAGTGTTGCTCAACATGCTAATAGCCATGATAAACAACTCCTATCAGG
AAATTGAGGAGGATGCAGATGTGGAATGGAAGTTCGCCCGAGCAAAACTCTGGCTGTCTTACTTTGATGAAGGAAGAACTCTACCTGCT
CCTTTTAATCTAGTGCCAAGTCCTAAATCATTTTATTATCTCATAATGAGAATCAAGATGTGCCTCATAAAACTCTGCAAATCTAAGGC
CAAAAGCTGTGAAAATGACCTTGAAATGGGCATGCTGAATTCCAAATTCAAGAAGACTCGCTACCAGGCTGGCATGAGGAATTCTGAAA
ATCTGACAGCAAATAACACTTTGAGCAAGCCCACCAGATACCAGAAAATCATGAAACGGCTCATAAAAAGATACGTCCTGAAAGCCCAG
GTGGACAGAGAAAATGACGAAGTCAATGAAGGCGAGCTGAAGGAAATCAAGCAAGATATCTCCAGCCTGCGCTATGAGCTTCTTGAGGA
AAAATCTCAAGCTACTGGTGAGCTGGCAGACCTGATTCAACAACTCAGCGAGAAGTTTGGAAAGAACTTAAACAAAGACCACCTGAGGG
TGAACAAGGGCAAAGACATTAAGCTTCCCGCCACCATGACCAGCAAGGTGTACGACCCCGAGCAGAGGAAGAGGATGATCACCGGCCCC
CAGTGGTGGGCCAGGTGCAAGCAGATGAACGTGCTGGACAGCTTCATCAACTACTACGACAGCGAGAAGCACGCCGAGAACGCCGTGAT
CTTCCTGCACGGCAACGCCACTAGCAGCTACCTGTGGAGGCACGTGGTGCCCCACATCGAGCCCGTGGCCAGGTGCATCATCCCCGATC
TGATCGGCATGGGCAAGAGCGGCAAGAGCGGCAACGGCAGCTACAGGCTGCTGGACCACTACAAGTACCTGACCGCCTGGTTCGAGCTC
CTGAACCTGCCCAAGAAGATCATCTTCGTGGGCCACGACTGGGGCGCCGCCCTGGCCTTCCACTACAGCTACGAGCACCAGGACAAGAT
CAAGGCCATCGTGCACGCCGAGAGCGTGGTGGACGTGATCGAGAGCTGGGACGAGTGGCCAGACATCGAGGAGGACATCGCCCTGATCA
AGAGCGAGGAGGGCGAGAAGATGGTGCTGGAGAACAACTTCTTCGTGGAGACCGTGCTGCCCAGCAAGATCATGAGAAAGCTGGAGCCC
GAGGAGTTCGCCGCCTACCTGGAGCCCTTCAAGGAGAAGGGCGAGGTGAGAAGACCCACCCTGAGCTGGCCCAGAGAGATCCCCCTGGT
GAAGGGCGGCAAGCCCGACGTGGTGCAGATCGTGAGAAACTACAACGCCTACCTGAGAGCCAGCGACGACCTGCCCAAGATGTTCATCG
AGAGCGACCCCGGCTTCTTCAGCAACGCCATCGTGGAGGGCGCCAAGAAGTTCCCCAACACCGAGTTCGTGAAGGTGAAGGGCCTGCAC
TTCAGCCAGGAGGACGCCCCCGACGAGATGGGCAAGTACATCAAGAGCTTCGTGGAGAGAGTGCTGAAGAACGAGCAGTAAGAATTCTC
TAGAGGGCCCGTTTAAACCCGCTGATCAGCCTCGACTGTGCCTTCTAGTTGCCAGCCATCTGTTGTTTGCCCCTCCCCCGTGCCTTCCT
TGACCCTGGAAGGTGCCACTCCCACTGTCCTTTCCTAATAAAATGAGGAAATTGCATCGCATTGTCTGAGTAGGTGTCATTCTATTCTG
GGGGGTGGGGTGGGGCAGGACAGCAAGGGGGAGGATTGGGAAGACAATAGCAGGCATGCTGGGGATGCGGTGGGCTCTATGGCTTCTGA
GGCGGAAAGAACCAGCTGGGGCTCTAGGGGGTATCCCCACGCGCCCTGTAGCGGCGCATTAAGCGCGGCGGGTGTGGTGGTTACGCGCA
GCGTGACCGCTACACTTGCCAGCGCCCTAGCGCCCGCTCCTTTCGCTTTCTTCCCTTCCTTTCTCGCCACGTTCGCCGGCTTTCCCCGT
CAAGCTCTAAATCGGGGCATCCCTTTAGGGTTCCGATTTAGTGCTTTACGGCACCTCGACCCCAAAAAACTTGATTAGGGTGATGGTTC
ACGTAGTGGGCCATCGCCCTGATAGACGGTTTTTCGCCCTTTGACGTTGGAGTCCACGTTCTTTAATAGTGGACTCTTGTTCCAAACTG
GAACAACACTCAACCCTATCTCGGTCTATTCTTTTGATTTATAAGGGATTTTGGGGATTTCGGCCTATTGGTTAAAAAATGAGCTGATT
TAACAAAAATTTAACGCGAATTAATTCTGTGGAATGTGTGTCAGTTAGGGTGTGGAAAGTCCCCAGGCTCCCCAGGCAGGCAGAAGTAT
GCAAAGCATGCATCTCAATTAGTCAGCAACCAGGTGTGGAAAGTCCCCAGGCTCCCCAGCAGGCAGAAGTATGCAAAGCATGCATCTCA
ATTAGTCAGCAACCATAGTCCCGCCCCTAACTCCGCCCATCCCGCCCCTAACTCCGCCCAGTTCCGCCCATTCTCCGCCCCATGGCTGA
CTAATTTTTTTTATTTATGCAGAGGCCGAGGCCGCCTCTGCCTCTGAGCTATTCCAGAAGTAGTGAGGAGGCTTTTTTGGAGGCCTAGG
CTTTTGCAAAAAGCTCCCGGGAGCTTGTATATCCATTTTCGGATCTGATCAGCACGTGTTGACAATTAATCATCGGCATAGTATATCGG
CATAGTATAATACGACAAGGTGAGGAACTAAACCATGGCCAAGTTGACCAGTGCCGTTCCGGTGCTCACCGCGCGCGACGTCGCCGGAG
CGGTCGAGTTCTGGACCGACCGGCTCGGGTTCTCCCGGGACTTCGTGGAGGACGACTTCGCCGGTGTGGTCCGGGACGACGTGACCCTG
TTCATCAGCGCGGTCCAGGACCAGGTGGTGCCGGACAACACCCTGGCCTGGGTGTGGGTGCGCGGCCTGGACGAGCTGTACGCCGAGTG
GTCGGAGGTCGTGTCCACGAACTTCCGGGACGCCTCCGGGCCGGCCATGACCGAGATCGGCGAGCAGCCGTGGGGGCGGGAGTTCGCCC
TGCGCGACCCGGCCGGCAACTGCGTGCACTTCGTGGCCGAGGAGCAGGACTGACACGTGCTACGAGATTTCGATTCCACCGCCGCCTTC
TATGAAAGGTTGGGCTTCGGAATCGTTTTCCGGGACGCCGGCTGGATGATCCTCCAGCGCGGGGATCTCATGCTGGAGTTCTTCGCCCA
CCCCAACTTGTTTATTGCAGCTTATAATGGTTACAAATAAAGCAATAGCATCACAAATTTCACAAATAAAGCATTTTTTTCACTGCATT
CTAGTTGTGGTTTGTCCAAACTCATCAATGTATCTTATCATGTCTGTATACCGTCGACCTCTAGCTAGAGCTTGGCGTAATCATGGTCA
TAGCTGTTTCCTGTGTGAAATTGTTATCCGCTCACAATTCCACACAACATACGAGCCGGAAGCATAAAGTGTAAAGCCTGGGGTGCCTA
ATGAGTGAGCTAACTCACATTAATTGCGTTGCGCTCACTGCCCGCTTTCCAGTCGGGAAACCTGTCGTGCCAGCTGCATTAATGAATCG
GCCAACGCGCGGGGAGAGGCGGTTTGCGTATTGGGCGCTCTTCCGCTTCCTCGCTCACTGACTCGCTGCGCTCGGTCGTTCGGCTGCGG
CGAGCGGTATCAGCTCACTCAAAGGCGGTAATACGGTTATCCACAGAATCAGGGGATAACGCAGGAAAGAACATGTGAGCAAAAGGCCA
GCAAAAGGCCAGGAACCGTAAAAAGGCCGCGTTGCTGGCGTTTTTCCATAGGCTCCGCCCCCCTGACGAGCATCACAAAAATCGACGCT
CAAGTCAGAGGTGGCGAAACCCGACAGGACTATAAAG

Sequence S2: DNA sequence of RlucII-TRPC7-GFP10 biosensor.

CTTATCCGGTAACTATCGTCTTGAGTCCAACCCGGTAAGACACGACTTATCGCCACTGGCAGCAGCCACTGGTAACAGGATTAGCAGAG
CGAGGTATGTAGGCGGTGCTACAGAGTTCTTGAAGTGGTGGCCTAACTACGGCTACACTAGAAGGACAGTATTTGGTATCTGCGCTCTG
CTGAAGCCAGTTACCTTCGGAAAAAGAGTTGGTAGCTCTTGATCCGGCAAACAAACCACCGCTGGTAGCGGTGGTTTTTTTGTTTGCAA
GCAGCAGATTACGCGCAGAAAAAAAGGATCTCAAGAAGATCCTTTGATCTTTT
CTACGGGGTCTGACGCTCAGTGGAACGAAAACTCACGTTAAGGGATTTTGGTCATGAGATTATCAAAAAGGATCTTCACCTAGATCCTT
TTAAATTAAAAATGAAGTTTTAAATCAATCTAAAGTATATATGAGTAAACTTGGTCTGACAGTTACCAATGCTTAATCAGTGAGGCACC
TATCTCAGCGATCTGTCTATTTCGTTCATCCATAGTTGCCTGACTCCCCGTCGTGTAGATAACTACGATACGGGAGGGCTTACCATCTG
GCCCCAGTGCTGCAATGATACCGCGAGACCCACGCTCACCGGCTCCAGATTTATCAGCAATAAACCAGCCAGCCGGAAGGGCCGAGCGC
AGAAGTGGTCCTGCAACTTTATCCGCCTCCATCCAGTCTATTAATTGTTGCCGGGAAGCTAGAGTAAGTAGTTCGCCAGTTAATAGTTT
GCGCAACGTTGTTGCCATTGCTACAGGCATCGTGGTGTCACGCTCGTCGTTTGGTATGGCTTCATTCAGCTCCGGTTCCCAACGATCAA
GGCGAGTTACATGATCCCCCATGTTGTGCAAAAAAGCGGTTAGCTCCTTCGGTCCTCCGATCGTTGTCAGAAGTAAGTTGGCCGCAGTG
TTATCACTCATGGTTATGGCAGCACTGCATAATTCTCTTACTGTCATGCCATCCGTAAGATGCTTTTCTGTGACTGGTGAGTACTCAAC
CAAGTCATTCTGAGAATAGTGTATGCGGCGACCGAGTTGCTCTTGCCCGGCGTCAATACGGGATAATACCGCGCCACATAGCAGAACTT
TAAAAGTGCTCATCATTGGAAAACGTTCTTCGGGGCGAAAACTCTCAAGGATCTTACCGCTGTTGAGATCCAGTTCGATGTAACCCACT
CGTGCACCCAACTGATCTTCAGCATCTTTTACTTTCACCAGCGTTTCTGGGTGAGCAAAAACAGGAAGGCAAAATGCCGCAAAAAAGGG
AATAAGGGCGACACGGAAATGTTGAATACTCATACTCTTCCTTTTTCAATATTATTGAAGCATTTATCAGGGTTATTGTCTCATGAGCG
GATACATATTTGAATGTATTTAGAAAAATAAACAAATAGGGGTTCCGCGCACATTTCCCCGAAAAGTGCCACCTGACGTCGACGGATCG
GGAGATCTCCCGATCCCCTATGGTCGACTCTCAGTACAATCTGCTCTGATGCCGCATAGTTAAGCCAGTATCTGCTCCCTGCTTGTGTG
TTGGAGGTCGCTGAGTAGTGCGCGAGCAAAATTTAAGCTACAACAAGGCAAGGCTTGACCGACAATTGCATGAAGAATCTGCTTAGGGT
TAGGCGTTTTGCGCTGCTTCGCGATGTACGGGCCAGATATACGCGTTGACATTGATTATTGACTAGTTATTAATAGTAATCAATTACGG
GGTCATTAGTTCATAGCCCATATATGGAGTTCCGCGTTACATAACTTACGGTAAATGGCCCGCCTGGCTGACCGCCCAACGACCCCCGC
CCATTGACGTCAATAATGACGTATGTTCCCATAGTAACGCCAATAGGGACTTTCCATTGACGTCAATGGGTGGACTATTTACGGTAAAC
TGCCCACTTGGCAGTACATCAAGTGTATCATATGCCAAGTACGCCCCCTATTGACGTCAATGACGGTAAATGGCCCGCCTGGCATTATG
CCCAGTACATGACCTTATGGGACTTTCCTACTTGGCAGTACATCTACGTATTAGTCATCGCTATTACCATGGTGATGCGGTTTTGGCAG
TACATCAATGGGCGTGGATAGCGGTTTGACTCACGGGGATTTCCAAGTCTCCACCCCATTGACGTCAATGGGAGTTTGTTTTGGCACCA
AAATCAACGGGACTTTCCAAAATGTCGTAACAACTCCGCCCCATTGACGCAAATGGGCGGTAGGCGTGTACGGTGGGAGGTCTATATAA
GCAGAGCTCTCTGGCTAACTAGAGAACCCACTGCTTACTGGCTTATCGAAATTAATACGACTCACTATAGGGAGACCCAAGCTGGCTAG
CCACCATGACCAGCAAGGTGTACGACCCCGAGCAGAGGAAGAGGATGATCACCGGCCCCCAGTGGTGGGCCAGGTGCAAGCAGATGAAC
GTGCTGGACAGCTTCATCAACTACTACGACAGCGAGAAGCACGCCGAGAACGCCGTGATCTTCCTGCACGGCAACGCCACTAGCAGCTA
CCTGTGGAGGCACGTGGTGCCCCACATCGAGCCCGTGGCCAGGTGCATCATCCCCGATCTGATCGGCATGGGCAAGAGCGGCAAGAGCG
GCAACGGCAGCTACAGGCTGCTGGACCACTACAAGTACCTGACCGCCTGGTTCGAGCTCCTGAACCTGCCCAAGAAGATCATCTTCGTG
GGCCACGACTGGGGCGCCGCCCTGGCCTTCCACTACAGCTACGAGCACCAGGACAAGATCAAGGCCATCGTGCACGCCGAGAGCGTGGT
GGACGTGATCGAGAGCTGGGACGAGTGGCCAGACATCGAGGAGGACATCGCCCTGATCAAGAGCGAGGAGGGCGAGAAGATGGTGCTGG
AGAACAACTTCTTCGTGGAGACCGTGCTGCCCAGCAAGATCATGAGAAAGCTGGAGCCCGAGGAGTTCGCCGCCTACCTGGAGCCCTTC
AAGGAGAAGGGCGAGGTGAGAAGACCCACCCTGAGCTGGCCCAGAGAGATCCCCCTGGTGAAGGGCGGCAAGCCCGACGTGGTGCAGAT
CGTGAGAAACTACAACGCCTACCTGAGAGCCAGCGACGACCTGCCCAAGATGTTCATCGAGAGCGACCCCGGCTTCTTCAGCAACGCCA
TCGTGGAGGGCGCCAAGAAGTTCCCCAACACCGAGTTCGTGAAGGTGAAGGGCCTGCACTTCAGCCAGGAGGACGCCCCCGACGAGATG
GGCAAGTACATCAAGAGCTTCGTGGAGAGAGTGCTGAAGAACGAGCAGGGATCCGCCGGTACCATGTTGAGGAACAGCACCTTCAAAAA
CATGCAGCGCCGGCACACAACGCTGAGGGAGAAGGGCCGTCGCCAGGCCATCCGGGGTCCCGCCTACATGTTCAACGAGAAGGGCACCA
GTCTGACGCCCGAGGAGGAGCGCTTCCTGGACTCGGCTGAGTATGGCAACATCCCGGTGGTCCGGAAAATGCTGGAGGAGTCCAAGACC
CTTAACTTCAACTGTGTGGACTACATGGGGCAGAACGCTCTGCAGCTGGCCGTGGGCAACGAGCACCTAGAGGTCACGGAGCTGCTGCT
GAAGAAGGAGAACCTGGCACGGGTGGGGGACGCGCTGCTGCTGGCCATCAGCAAGGGCTATGTGCGCATCGTGGAGGCCATCCTCAACC
ACCCGGCCTTCGCGCAGGGCCAGCGCCTGACGCTCAGCCCGCTGGAACAGGAGCTGCGCGACGACGACTTCTATGCCTACGACGAGGAC
GGCACGCGCTTCTCCCACGACATCACGCCCATCATCCTGGCGGCGCACTGCCAGGAGTATGAGATCGTGCACATCCTGCTGCTCAAGGG
CGCCCGCATCGAGCGGCCCCACGACTACTTCTGCAAGTGCAATGAGTGCACCGAGAAACAGCGGAAAGACTCCTTCAGCCACTCGCGCT
CGCGCATGAACGCCTACAAAGGACTGGCGAGTGCTGCCTACTTGTCCCTGTCCAGCGAAGACCCTGTCCTCACCGCCCTGGAGCTCAGC
AACGAGTTAGCCAGACTAGCCAACATTGAGACTGAATTTAAGAACGATTACAGGAAGTTATCTATGCAATGCAAGGATTTTGTAGTGGG
CGTGCTGGACCTGTGCCGAGACACAGAAGAGGTGGAAGCAATTTTAAACGGTGATGTGAACTTCCAAGTCTGGTCCGACCACCACCGTC
CAAGTCTGAGCCGGATCAAACTCGCCATTAAATATGAAGTCAAGAAGTTCGTTGCTCATCCTAACTGTCAGCAGCAATTGCTTACCATG
TGGTATGAAAATCTCTCAGGCTTACGTCAACAGTCTATCGCTGTGAAATTCCTGGCTGTCTTTGGAGTCTCCATAGGCCTCCCTTTTCT
CGCCATAGCCTATTGGATTGCTCCGTGCAGCAAGCTAGGACGAACCCTGAGGAGCCCTTTCATGAAGTTTGTAGCTCATGCAGTTTCTT
TTACAATCTTCTTGGGATTATTAGTTGTGAATGCATCTGACCGATTTGAAGGTGTTAAAACCCTGCCAAACGAAACCTTCACAGACTAC
CCAAAACAAATCTTCAGAGTGAAAACCACACAGTTCTCCTGGACAGAAATGCTCATTATGAAGTGGGTCTTAGGAATGATTTGGTCCGA
ATGCAAGGAAATCTGGGAGGAGGGGCCACGGGAGTACGTGCTGCACTTGTGGAACCTGCTAGATTTCGGGATGCTGTCCATCTTCGTGG
CCTCCTTCACAGCACGCTTCATGGCCTTCCTGAAGGCCACGGAGGCACAGCTGTACGTGGACCAGCACGTGCAGGACGACACGCTGCAC
AATGTCTCGCTTCCGCCGGAAGTGGCATACTTCACCTACGCCAGGGACAAGTGGTGGCCTTCAGACCCTCAGATCATATCGGAAGGGCT
CTACGCGATAGCCGTCGTGCTGAGCTTCTCTCGCATTGCATACATTCTGCCAGCCAACGAGAGTTTTGGGCCCCTGCAGATCTCGCTAG
GGAGAACTGTGAAAGATATCTTCAAGTTCATGGTCATTTTCATCATGGTATTTGTGGCCTTCATGATTGGGATGTTCAACCTGTACTCT

TACTACCGAGGTGCCAAATACAACCCAGCGTTTACAACGGTTGAAGAAAGTTTTAAAACTTTGTTTTGGTCCATATTCGGCTTATCTGA
AGTAATCTCAGTGGTGCTGAAATACGACCACAAATTCATCGAGAACATTGGCTACGTTCTCTACGGCGTTTATAACGTCACCATGGTGG
TAGTGTTGCTCAACATGCTAATAGCCATGATAAACAACTCCTATCAGGAAATTGAGGAGGATGCAGATGTGGAATGGAAGTTCGCCCGA
GCAAAACTCTGGCTGTCTTACTTTGATGAAGGAAGAACTCTACCTGCTCCTTTTAATCTAGTGCCAAGTCCTAAATCATTTTATTATCT
CATAATGAGAATCAAGATGTGCCTCATAAAACTCTGCAAATCTAAGGCCAAAAGCTGTGAAAATGACCTTGAAATGGGCATGCTGAATT
CCAAATTCAAGAAGACTCGCTACCAGGCTGGCATGAGGAATTCTGAAAATCTGACAGCAAATAACACTTTGAGCAAGCCCACCAGATAC
CAGAAAATCATGAAACGGCTCATAAAAAGATACGTCCTGAAAGCCCAGGTGGACAGAGAAAATGACGAAGTCAATGAAGGCGAGCTGAA
GGAAATCAAGCAAGATATCTCCAGCCTGCGCTATGAGCTTCTTGAGGAAAAAATCTCAAGCTACTGGTGAGCTGGCAGACCTGATTCAAC
AACTCAGCGAGAAGTTTGGAAAGAACTTAAACAAAGACCACCTGAGGGTGAACAAGGGCAAAGACATTAAGCTTCCCGCCACCATGGTG
AGCAAGGGCGAGGAGCTGTTCACCGGGGTGGTGCCCATCCTGGTCGAGCTGGACGGCGACGTAAACGGCCACAAGTTCAGCGTGTCCGG
CGAGGGCGAGGGCGATGCCACCTACGGCAAGCTGACCCTGAAGTTCATCTGCACCACCGGCAAGCTGCCCGTGCCCTGGCCCACCCTCG
TGACCACCCTGAGCTACGGCGTGCAGTGCTTCAGCCGCTACCCCGACCACATGAAGCAGCACGACTTCTTCAAGTCCGCCATGCCCGAA
GGCTACGTCCAGGAGCGCACCATCTTCTTCAAGGACGACGGCAACTACAAGACCCGCGCCGAGGTGAAGTTCGAGGGCGACACCCTGGT
GAACCGCATCGAGCTGAAGGGCATCGACTTCAAGGAGGACGGCAACATCCTGGGGCACAAGCTGGAGTACAACTACAACCCCCACAACG
TCTATATCATGGCCGACAAGCAGAAGAACGGCATCAAGGTGAACTTCAAGATCCGCCACAACATCGAGGACGGCAGCGTGCAGCTCGCC
GACCACTACCAGCAGAACACCCCCATCGGCGACGGCCCCGTGCTGCTGCCCGACAACCACTACCTGTTCACCCAGTCCGCCCTGAGCAA
AGACCCCAACGAGAAGCGCGATCACATGGTCCTGCTGGAGTTCGTGACCGCCGCCGGGATCACTCTCGGCATGGACGAGCTGTACAAGT
GGAGCCACCCGCAGTTCGAAAAATAACTCGAGTCTAGAGGGCCCGTTTAAACCCGCTGATCAGCCTCGACTGTGCCTTCTAGTTGCCAG
CCATCTGTTGTTTGCCCCTCCCCCGTGCCTTCCTTGACCCTGGAAGGTGCCACTCCCACTGTCCTTTCCTAATAAAATGAGGAAATTGC
ATCGCATTGTCTGAGTAGGTGTCATTCTATTCTGGGGGGTGGGGTGGGGCAGGACAGCAAGGGGGAGGATTGGGAAGACAATAGCAGGC
ATGCTGGGGATGCGGTGGGCTCTATGGCTTCTGAGGCGGAAAGAACCAGCTGGGGCTCTAGGGGGTATCCCCACGCGCCCTGTAGCGGC
GCATTAAGCGCGGCGGGTGTGGTGGTTACGCGCAGCGTGACCGCTACACTTGCCAGCGCCCTAGCGCCCGCTCCTTTCGCTTTCTTCCC
TTCCTTTCTCGCCACGTTCGCCGGCTTTCCCCGTCAAGCTCTAAATCGGGGCATCCCTTTAGGGTTCCGATTTAGTGCTTTACGGCACC
TCGACCCCAAAAAACTTGATTAGGGTGATGGTTCACGTAGTGGGCCATCGCCCTGATAGACGGTTTTTCGCCCTTTGACGTTGGAGTCC
ACGTTCTTTAATAGTGGACTCTTGTTCCAAACTGGAACAACACTCAACCCTATCTCGGTCTATTCTTTTGATTTATAAGGGATTTTGGG
GATTTCGGCCTATTGGTTAAAAAATGAGCTGATTTAACAAAAATTTAACGCGAATTAATTCTGTGGAATGTGTGTCAGTTAGGGTGTGG
AAAGTCCCCAGGCTCCCCAGGCAGGCAGAAGTATGCAAAGCATGCATCTCAATTAGTCAGCAACCAGGTGTGGAAAGTCCCCAGGCTCC
CCAGCAGGCAGAAGTATGCAAAGCATGCATCTCAATTAGTCAGCAACCATAGTCCCGCCCCTAACTCCGCCCATCCCGCCCCTAACTCC
GCCCAGTTCCGCCCATTCTCCGCCCCATGGCTGACTAATTTTTTTTATTTATGCAGAGGCCGAGGCCGCCTCTGCCTCTGAGCTATTCC
AGAAGTAGTGAGGAGGCTTTTTTGGAGGCCTAGGCTTTTGCAAAAAGCTCCCGGGAGCTTGTATATCCATTTTCGGATCTGATCAGCAC
GTGTTGACAATTAATCATCGGCATAGTATATCGGCATAGTATAATACGACAAGGTGAGGAACTAAACCATGGCCAAGTTGACCAGTGCC
GTTCCGGTGCTCACCGCGCGCGACGTCGCCGGAGCGGTCGAGTTCTGGACCGACCGGCTCGGGTTCTCCCGGGACTTCGTGGAGGACGA
CTTCGCCGGTGTGGTCCGGGACGACGTGACCCTGTTCATCAGCGCGGTCCAGGACCAGGTGGTGCCGGACAACACCCTGGCCTGGGTGT
GGGTGCGCGGCCTGGACGAGCTGTACGCCGAGTGGTCGGAGGTCGTGTCCACGAACTTCCGGGACGCCTCCGGGCCGGCCATGACCGAG
ATCGGCGAGCAGCCGTGGGGGCGGGAGTTCGCCCTGCGCGACCCGGCCGGCAACTGCGTGCACTTCGTGGCCGAGGAGCAGGACTGACA
CGTGCTACGAGATTTCGATTCCACCGCCGCCTTCTATGAAAGGTTGGGCTTCGGAATCGTTTTCCGGGACGCCGGCTGGATGATCCTCC
AGCGCGGGGATCTCATGCTGGAGTTCTTCGCCCACCCCAACTTGTTTATTGCAGCTTATAATGGTTACAAATAAAGCAATAGCATCACA
AATTTCACAAATAAAGCATTTTTTTCACTGCATTCTAGTTGTGGTTTGTCCAAACTCATCAATGTATCTTATCATGTCTGTATACCGTC
GACCTCTAGCTAGAGCTTGGCGTAATCATGGTCATAGCTGTTTCCTGTGTGAAATTGTTATCCGCTCACAATTCCACACAACATACGAG
CCGGAAGCATAAAGTGTAAAGCCTGGGGTGCCTAATGAGTGAGCTAACTCACATTAATTGCGTTGCGCTCACTGCCCGCTTTCCAGTCG
GGAAACCTGTCGTGCCAGCTGCATTAATGAATCGGCCAACGCGCGGGGAGAGGCGGTTTGCGTATTGGGCGCTCTTCCGCTTCCTCGCT
CACTGACTCGCTGCGCTCGGTCGTTCGGCTGCGGCGAGCGGTATCAGCTCACTCAAAGGCGGTAATACGGTTATCCACAGAATCAGGGG
ATAACGCAGGAAAGAACATGTGAGCAAAAGGCCAGCAAAAGGCCAGGAACCGTAAAAAGGCCGCGTTGCTGGCGTTTTTCCATAGGCTC
CGCCCCCCTGACGAGCATCACAAAAATCGACGCTCAAGTCAGAGGTGGCGAAACCCGACAGGACTATAAAGATACCAGGCGTTTCCCCC
TGGAAGCTCCCTCGTGCGCTCTCCTGTTCCGACCCTGCCGCTTACCGGATACCTGTCCGCCTTTCTCCCTTCGGGAAGCGTGGCGCTTT
CTCAATGCTCACGCTGTAGGTATCTCAGTTCGGTGTAGGTCGTTCGCTCCAAGCTGGGCTGTGTGCACGAACCCCCCGTTCAGCCCGAC
CGCTGCGC
